# A Laing distal myopathy–associated proline substitution in the **β**-myosin rod perturbs myosin cross-bridging activity

**DOI:** 10.1172/JCI172599

**Published:** 2024-05-01

**Authors:** Massimo Buvoli, Genevieve C.K. Wilson, Ada Buvoli, Jack F. Gugel, Abbi Hau, Carsten G. Bönnemann, Carmen Paradas, David M. Ryba, Kathleen C. Woulfe, Lori A. Walker, Tommaso Buvoli, Julien Ochala, Leslie A. Leinwand

**Affiliations:** 1 Department of Molecular, Cellular and Developmental Biology, and; 2 BioFrontiers Institute, Department of Molecular, Cellular and Developmental Biology, University of Colorado, Boulder, Colorado, USA.; 3 Centre of Human and Applied Physiological Sciences, School of Basic and Medical Biosciences, Faculty of Life Sciences and Medicine, and; 4 Randall Centre for Cell and Molecular Biophysics, School of Basic and Medical Biosciences, Faculty of Life Sciences and Medicine, Guy’s Campus, King’s College London, London, United Kingdom.; 5 Neuromuscular and Neurogenetic Disorders of Childhood Section, National Institute of Neurological Disorders and Stroke (NINDS), NIH, Bethesda, Maryland, USA.; 6 Neuromuscular Unit, Department of Neurology, Instituto de Biomedicina de Sevilla (IBIS), Hospital Universitario Virgen del Rocío/CSIC/Universidad de Sevilla, Sevilla, Spain.; 7 Bristol Myers Squibb, Brisbane, California, USA.; 8 Division of Cardiology, Department of Medicine, University of Colorado, Denver, Colorado, USA.; 9 Department of Mathematics, Tulane University, New Orleans, Louisiana, USA.; 10 Department of Biomedical Sciences, University of Copenhagen, Copenhagen, Denmark.

**Keywords:** Muscle biology, Molecular pathology, Mouse models, Muscle

## Abstract

Proline substitutions within the coiled-coil rod region of the β-myosin gene (MYH7) are the predominant mutations causing Laing distal myopathy (MPD1), an autosomal dominant disorder characterized by progressive weakness of distal/proximal muscles. We report that the MDP1 mutation R1500P, studied in what we believe to be the first mouse model for the disease, adversely affected myosin motor activity despite being in the structural rod domain that directs thick filament assembly. Contractility experiments carried out on isolated mutant muscles, myofibrils, and myofibers identified muscle fatigue and weakness phenotypes, an increased rate of actin-myosin detachment, and a conformational shift of the myosin heads toward the more reactive disordered relaxed (DRX) state, causing hypercontractility and greater ATP consumption. Similarly, molecular analysis of muscle biopsies from patients with MPD1 revealed a significant increase in sarcomeric DRX content, as observed in a subset of myosin motor domain mutations causing hypertrophic cardiomyopathy. Finally, oral administration of MYK-581, a small molecule that decreases the population of heads in the DRX configuration, significantly improved the limited running capacity of the R1500P-transgenic mice and corrected the increased DRX state of the myofibrils from patients. These studies provide evidence of the molecular pathogenesis of proline rod mutations and lay the groundwork for the therapeutic advancement of myosin modulators.

## Abstract 2

Proline substitutions within the coiled-coil of β-myosin cause Laing distal myopathy by perturbing myosin cross-bridging activity.

## Graphical abstract

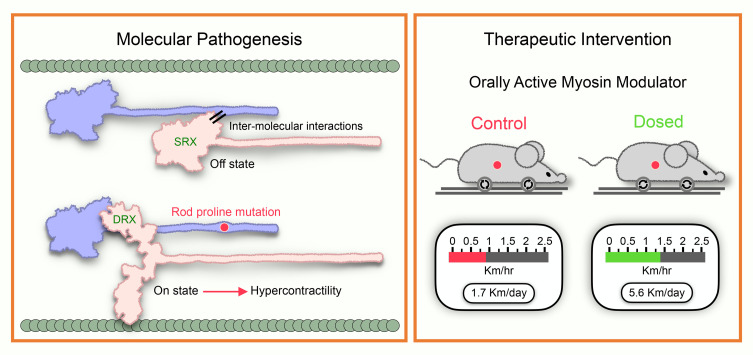

## Introduction

Laing distal myopathy, also called myopathy distal type 1 (MPD1), is an autosomal dominant disease with variable onset and phenotypic severity. Typically, MPD1 begins with weakness of the anterior compartment of the lower leg, which ranges from mild to severe and then progresses to other skeletal muscle groups, often showing variable histology (1–3). Currently, there are no approved treatments for the cause of the disease.

MPD1-causative mutations have been mapped to the *MYH7* gene that encodes the β-myosin heavy chain, which is expressed in both the human heart and in type 1 slow skeletal muscle fibers (4). Of interest, only a small number of patients with MPD1 develop concomitant cardiomyopathy, despite β-myosin being the predominant myosin in the human heart (1, 5–8). More than 900 pathogenic mutations causing different cardiac and skeletal myopathies have been identified in both the N-terminal motor domain and the coiled-coil rod domain of the molecule (Human Gene Mutation Database [HGMD]). However, the majority of MPD1 mutations are located in the light meromyosin (LMM) region of the rod domain that controls myosin assembly into the thick filaments (7), although only a small number of them are located in the motor domain (9, 10). MPD1 rod mutations are primarily codon deletions and missense mutations that introduce a proline residue. Both of these genetic defects are predicted to negatively affect the structure of the myosin coiled-coil. In particular, proline residues located in the middle of α-helices introduce a kink that could locally perturb the myosin coiled-coil structure (11).

The biological effects of a subset of MPD1 mutations have been characterized in different cell systems (12, 13). These studies have shown that proline rod mutations do not prevent incorporation of mutant myosin into the sarcomere (12) and therefore do not hinder formation of the rod coiled-coil structure as originally proposed (14). However, they induce formation of myosin cytoplasmic aggregates (12) and cause aberrant myosin packing in thick filaments (15). Interestingly, a dominant skeletal muscle myopathy caused by a mutation in MYH7 that induces a MPD1-like phenotype has been reported in pigs (16). More recently, a *Drosophila melanogaster* model for MPD1 has also been successfully established (17). In spite of these different investigations, the pathogenic mechanisms of MPD1 mutations remain unclear. While numerous genetic mouse models have been developed for studying mutations in the MYH7 motor domain that cause hypertrophic or dilated cardiomyopathy (18), no mammalian genetic models have yet been reported for examining the effects of myopathy-causing mutations in the rod domain.

Here, we report that expression of the R1500P-mutant myosin previously identified in patients with MPD1 (14) affects mouse muscle histology and performance. Our data suggest a shift in the myosin energetic state, a finding also observed in a group of hypertrophic cardiomyopathy mutations located in the motor domain, as the primary cause of disease pathogenesis. The finding that the exercise capacity of the R1500P-transgenic animal significantly improved after treatment with a myosin modulator suggests the first potential therapeutic treatment for patients with MPD1.

## Results

### Establishment of a mouse model of MPD1.

**Figure 1:**
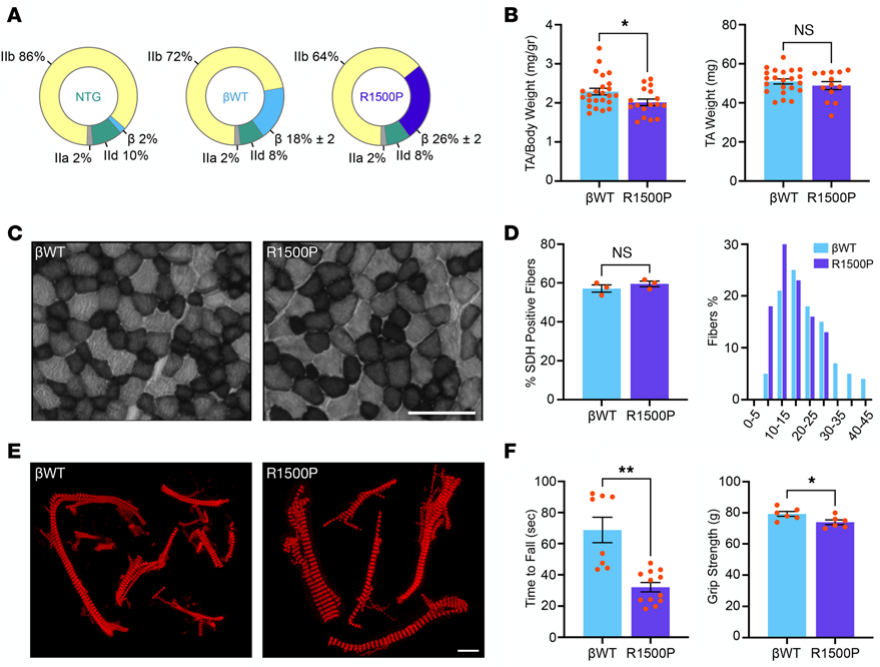
Transgenic mice expressing the myosin mutant R1500P have reduced muscle strength. (**A**) Pie chart shows the relative percentage of the different myosin isoforms expressed in TA muscles of NTG mice, transgenic mice expressing WT MYH7 (βWT), and mice expressing mutant MYH7 (R1500P), quantified by myosin unique peptide proteomics. Six- to 7-month-old male mice were studied (*n* = 3 mice/group). (**B**) Average ratio of TA muscle/body weight (left) and TA muscle weight (right); Six- to 10-month-old male mice were studied (*n* = 20 mice/group). (**C**) Representative SDH activity of TA muscle cross-sections isolated from 8-month-old male βWT (left) and R1500P (right) animals. Scale bar: 200 μm. (**D**) Quantification of SDH-positive fibers (left), and fiber CSA, reported in μm^2^ divided by 100 (right). Representative images of sections (fields) used in the CSA quantification are shown in **E** and Supplemental Figure 2. (**E**) Myofibrils from TA muscles from 6-month-old male βWT (left) and R1500P (right) mice were prepared and immunostained with the Myc antibody. Samples were imaged on a confocal spinning-disk microscope (Nikon Ti-E; ×100 silicon immersion objective). Scale bar: 5 μm. (**F**) Four-limb hanging (left) and grip strength (right) tests. The hanging time for each animal was calculated after 3 trials. Eight-month-old male mice were used (*n* = 6 mice/group). Measurements of forelimb grip strength were carried out with a computerized grip strength meter. Seven- to 8-month-old male mice (*n* = 6 mice/group). All data are expressed as the mean ± SEM. *P < 0.05 and **P < 0.01, by 2-tailed, unpaired Student’s *t* test with Welch’s correction.

To create a mouse that models MPD1, we generated animals expressing the β-myosin R1500P mutation under the control of the well-characterized muscle creatine kinase (MCK) promoter (19–21). We used this strategy to circumvent the very low abundance of slow type I fibers in mice compared with humans. As a control, we also created mice expressing WT βMyHC. To monitor transgene expression, a Myc epitope was inserted at the end of the myosin coiled-coil region; as previously reported, the addition of the tag does not interfere with myosin sarcomeric function (22). We obtained 3 transgenic βWT/mutant lines expressing similar amounts of the 2 transgenes in both tibialis anterior (TA) and medial gastrocnemius muscles (Supplemental Figure 1A; supplemental material available online with this article; https://doi.org/10.1172/JCI172599DS1). Moreover, as originally described (23), we also found that the 4,800 bp MCK promoter fragment preferentially drove expression of the transgenes in fast skeletal muscle with minimal expression in heart and slow fibers (Supplemental Figure 1A). Quantification of myosin composition in TA transgenic muscles by mass spectrometry analysis of isoform-specific peptides showed a difference of approximately 8% in relative expression of the βWT and R1500P myosins. Notably, the presence of both transgenes caused a proportional reduction in the levels of the myosin isoform IIb without affecting the expression of IIa or IId (Figure 1A). Thus, the total amount of myosin did not differ between transgenic and nontransgenic (NTG) mice.

### The R1500P mutation causes skeletal myopathy.

In patients with MDP1, the consistent muscle weakness phenotype is not usually accompanied by representative histological signatures of the disease. The most common muscle abnormalities include variation in muscle fiber size, fiber type 1 predominance with small type 1 fibers and large type 2 fibers, coexpression of slow and fast myosin, type 1 fiber atrophy/hypotrophy, and core/multicore pathology (1, 24). In our mouse model, we found that, although expression of the R1500P mutant significantly decreased the TA/body weight ratio (Figure 1B, left), it did not change the muscle weight of the mutant TA muscle (Figure 1B, right). Furthermore, histological analysis of βWT and R1500P TA muscle enzymatically stained for the mitochondrial enzyme succinate dehydrogenase (SDH) showed no significant difference in the percentage of positive fibers (Figure 1, C and D, left). However, while the proportion of fast versus slow muscle fibers was unchanged, measurement of the fiber cross-sectional area (CSA) showed that R1500P muscle had a higher proportion of smaller muscle fibers than did the βWT control (Figure 1D, right, and Supplemental Figure 2). Transmission electron microscopy (TEM) analysis revealed that the integrity of the sarcomere was not affected in TA muscle. However, we observed ultrastructural changes in the sarcoplasmic reticulum (SR) and t-tubules in the R1500P-transgenic animals. Although βWT muscles showed a normal pattern of tightly wound SR networks with accompanying t-tubule triads, R1500P muscles had irregular and enlarged SRs, with associated t-tubules having mild-to-severe dilation of the triad structure (Supplemental Figure 3A). A comparable phenotype has been described in some patients with MDP1 (1). Although mitochondrial abnormalities are considered a hallmark of core myopathies, only 1 study has reported mitochondrial proliferation in patients with MDP1 (1). Nonetheless, we performed mitochondrial proteomics analysis on our mass spectrometry data obtained from βWT and R1500P muscles. This survey did not reveal any significant changes in the abundance of either mitochondria- or nucleus-encoded proteins, with the exception of LETM1, which is required for mitochondrial homeostasis and cell viability (25) and was upregulated in the R1500P samples (Supplemental Figure 3, B and C). DRP1 and OpaI proteins, which are key proteins controlling, respectively, mitochondrial fission (critical for mitochondrial DNA [mtDNA] copy number maintenance) and fusion, were not activated in the R1500P-transgenic muscle. Moreover, the levels of proteins considered to have a good correlation with mtDNA copy number, such as the electron transport protein cluster and TFAM, a key component of mitochondria nucleoids (26), were normal. Thus, considering these results, we believe that mitochondria did not substantially contribute to the phenotype observed in the R1500P mice at the time of our assessment.

As previously observed in C_2_C_12_ myogenic cells (12), we found that the R1500P mutation did not prevent proper and efficient incorporation of the myosin mutant in the transgenic TA thick filaments (Figure 1E). To evaluate whether the R1500P mutation impairs muscle force generation in vivo, mice were subjected to 4-limb hanging and forelimb grip strength tests. While the first assay measured the falling delay of a mouse from a suspended wire mesh screen after exhaustion, the second one determined the maximum force generated by an animal being held by its forelimbs on to a metal rod (27). Both these muscle assessments revealed a significant decrease in sustained muscle tension and force output in R1500P animals when compared with βWT controls (Figure 1F). Thus, as seen in patients with MPD1, murine expression of the R1500P myosin mutant negatively affected muscle strength.

### The R1500P mutation induces muscle hypercontractility and fatigue.

**Figure 2:**
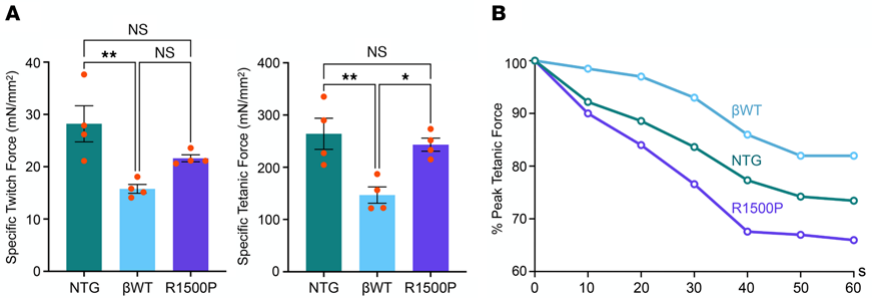
Expression of the myosin mutant R1500P alters skeletal muscle performance. EDL muscles isolated from NTG, βWT, and R1500P mice were subjected to an ex vivo contractility assay. (**A**) Comparison of specific twitch force and tetanic force. Six-month-old male mice (*n* = 4/mouse group) were used. Data are expressed as the mean ± SEM. *P < 0.05 and **P < 0.01, by 1-way ANOVA with Bonferroni’s multiple-comparison post hoc test. (**B**) Influence of intermittent tetanic stimulation on fatigue. The tetanic stimulation protocol was 100 Hz for 500 ms, once every second for 60 seconds (representative plot of 3 independent experiments). Six-month-old male mice were used.

Informative parameters concerning muscle force, contractile kinetics, and fatigability can be measured in isolated skeletal muscle. Thus, we next assessed the ex vivo properties of the extensor digitorum longus (EDL) muscle, analyzed instead of the TA muscle, since an intact muscle-tendon complex is required for the assay (28). As seen in TA, both transgenes were expressed and incorporated into EDL thick filaments (Supplemental Figure 1B). We found that βWT EDL had a significant drop in both twitch and tetanic amplitude (~1.8-fold each) (Figure 2A). This result was not unexpected, since expression of the slow β-myosin transgenes caused a proportional reduction in the levels of fast IIb-myosin (Figure 1A). As a consequence of this induced remodeling, the greater presence of slow fibers in transgenic muscles retunes their performance and decreases their power output, as reported in single-skinned muscle fibers from rat EDL and soleus (29) and in muscle fibers isolated from endurance athletes (30). In contrast, we found that the R1500P tetanic force was increased 1.6-fold compared with the force measured in βWT muscle, suggesting a hypercontractile phenotype that counteracted the drop in force generation observed in the βWT mice (Figure 2A). Finally, the twitch/tetanus ratio was similar for NTG, βWT, and R1500P muscles (Supplemental Figure 4). To assess whether expression of the R1500P mutant could induce fatigue, in addition to the observed muscle weakness in 4-limb hanging and grip strength tests, we next subjected βWT and mutant EDL muscles to a fatigue protocol measuring the correlation between the relative peak tetanic force (percentage) and time F(*t*) (31). A decline of the R1500P EDL peak force to approximately 90% and approximately 85% by the 10th and 29th tetanus stimuli compared with the more stable percentage measured for the βWT muscle (Figure 2B) confirmed that the myosin mutant provoked fatigue and suggested that in patients with MPD1, muscle fatigue and weakness can occur concurrently, as reported, for example, in patients with myasthenia gravis (32). Moreover, the decay trace of the NTG peak force, which fell between the βWT and the R1500P traces, indicates that expression of the slow βWT myosin in the transgenic muscles made them less susceptible to fatigue, as naturally seen during the muscle adaptation of endurance athletes (33).

### The R1500P mutation engenders faster cross-bridge relaxation.

To broaden our biophysical characterization of muscles expressing the R1500P mutation, we next measured the cross-bridge kinetics of myofibril preparations isolated from transgenic TA muscles. Mounted myofibrils were activated and relaxed by rapidly switching between 2 flowing solutions of pCa 4.5 and pCa 9.0. While activation (ACT) is described by a single exponential (EXP) function with a constant rate *k*_ACT_, relaxation (REL) of myofibrils follows a biphasic state, i.e., an initial slow linear (LIN) phase followed by a faster exponential decay. While the rate and duration of the slow phase (*k*_REL,LIN_ and *t*_REL,LIN_) is a slow force decay, with the sarcomere remaining isometric and mainly determined by cross-bridge detachment, the final exponential phase of force decline, defined by the fast rate constant *k*_REL,EXP_, is mainly determined by intersarcomere dynamics.

**Figure 3:**
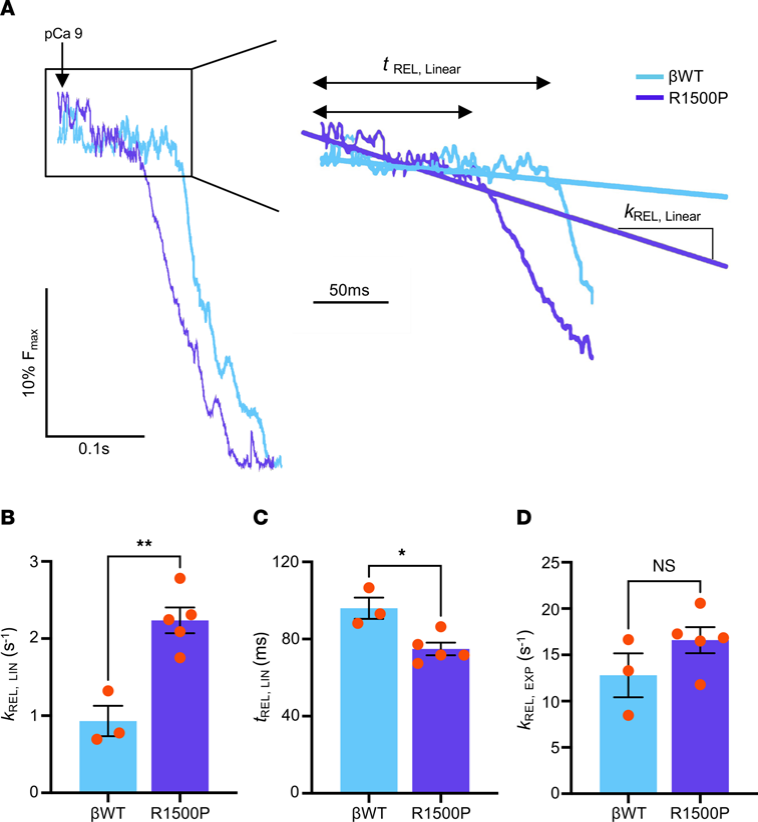
Myofibrils containing the myosin mutant R1500P show faster cross-bridge detachment under isometric conditions. (**A**) Representative trace of linear and exponential phase relaxation of myofibrils isolated from TA muscles. (**B**) Rate constant (*k*_REL,LIN_); (**C**) duration of the early linear phase of relaxation (*t*_REL,LIN_); and (**D**) rate constant of the final exponential phase of force decline (*k*_REL,EXP_) measured from βWT and R1500P myofibrils. The activity of 4–6 myofibrils/muscle was averaged. Six-month-old male mice were used (βWT, *n* = 3; R1500P, *n* = 5). Data are expressed as the mean ± SEM. *P < 0.05 and **P < 0.01, by 2-tailed, unpaired Student’s *t* test with Welch’s correction. See Supplemental Figure 6 for individual βWT and R1500P myofibril measurements.

Although we did not detect changes in the activation kinetics, *k*_ACT_ or *k*_TR_ or rate constant of tension redevelopment (TR) (Supplemental Figure 5), we found that the rate of slow-phase relaxation (*k*_REL,LIN_) was significantly faster in R1500P myofibrils than in βWT control myofibrils (Figure 3, A and B, and Supplemental Figure 6A), whereas the duration of slow-phase relaxation (*t*_REL,LIN_) was significantly decreased (Figure 3C and Supplemental Figure 6B). In contrast, the rate of the rapid exponential phase of relaxation (*k*_REL,EXP_) was unchanged (Figure 3D and Supplemental Figure 6C). Of note, faster cross-bridge detachment accompanied by hypercontractility caused by a higher number of myosin heads in the disordered relaxed (DRX) state have been reported for the β-MYH7 mutations R403Q and P710R, which cause hypertrophic cardiomyopathy (HCM) (34–36).

### The proportion of myosin molecules in the DRX state is increased in R1500P myofibrils.

**Figure 4:**
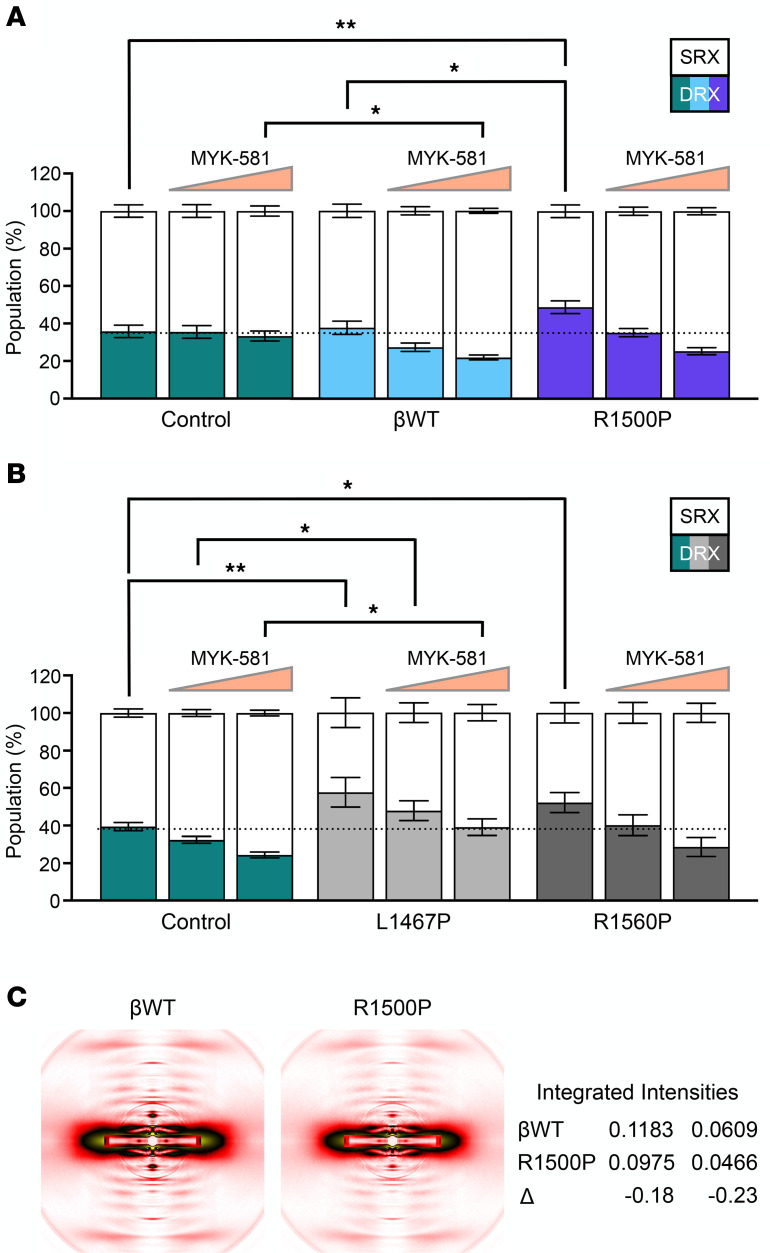
The R1500P mutation changes myosin enzymatic activity as well as the thick filament structure. Percentage of myosin molecules in the SRX or DRX state measured by Mant-ATP chase assay. (**A**) Analysis of purified muscle fibers from 6-month-old male βWT and R1500P mice. Control mice, *n* = 10; βWT mice, *n* = 11; R1500P mice, *n* = 12. (**B**) Analysis carried of biopsy specimens from patients with distal myopathy caused by the MYH7 missense mutations L1467P (35-year-old female) and R1560P (52-year-old male). Controls correspond to the mouse TA and human vastus lateralis muscles lacking or having type 1 fibers, respectively. Measurements were recorded in the absence or presence of MYK-581 (0.3 μM and 1 μM). Control mice, *n* = 17; L1467P mice, *n* = 4; R1560P mice, *n* = 6. See Supplemental Figure 7 for relative DRX scatter plots. Data are expressed as the mean ± SEM. *P < 0.05 and **P < 0.01, by 2-way ANOVA (genotype × drug) with Bonferroni’s multiple-comparison post hoc test. (**C**) MM reflections recorded from x-ray diffraction experiments carried out on βWT and R1500P purified fibers that were immersed in relaxing buffer, mounted in a specific chamber, and examined. All the MM1 and MM2 integrated intensities were normalized to the sixth actin layer line intensity.

Considering the unforeseen long-range effects of the R1500P rod mutation on actin-myosin kinetics, we next probed whether the presence of the mutant myosin in the thick filaments could alter the proportion of myosin molecules in DRX and super-relaxed (SRX) conformations, as found for the HCM mutations cited above. It is well known that myosin heads in the DRX conformation have an ATPase activity that is 5 times greater than that in heads in the SRX configuration (37). Thus, in relaxed muscle fibers, the DRX/SRX ratio, which correlates with the rate of ATP consumption, can be determined by measuring ATP utilization and decay of a fluorescent, nonhydrolysable ATP (Mant-ATP) (38). This analysis showed that R1500P single myofibers had a significantly higher fraction of myosin heads in the DRX conformation (48.75%), with a corresponding lower proportion of heads in the SRX conformation, compared with the control groups (~37.7% and 35.8% for control and βWT groups, respectively) (Figure 4A). To substantiate this finding, we next carried out a dose-response experiment with the mavacamten-like myosin inhibitor MYK-581, which reduces myosin’s ATPase rate by increasing the number of heads in SRX conformation (39). MYK-581 is a specific modulator of the 2 cardiac myosin heavy chains α and β, showing low affinity for the other myosin isoforms present in the skeletal muscles. We observed an inverse dose-response relationship between MYK-581 and myosin heads in DRX conformations, as well as a related direct relationship with heads in SRX conformations (Figure 4A). Notably, we obtained comparable results when the DRX/SRX analysis was performed on myofibers isolated from patients with MPD1 with proline substitutions located in the LMM region of the rod (Figure 4B, see also Methods). Thus, even in the human muscle context, pathogenic proline mutations located in the myosin coiled-coil domain raised the ratio of myosin heads in the DRX versus the SRX conformation. Most significantly, by restoring the DRX/SRX ratio to physiological levels, MYK-581 shows potential therapeutic efficacy for the treatment of MPD1.

### x-ray diffraction patterns of R1500P myofibrils reveal a decrease in the intensity of the first and second myosin meridional reflections.

To gather a deeper understanding of the molecular effects of the R1500P mutation on sarcomeric architecture, we then subjected βWT and R1500P myofibrils to low-angle x-rays, a technique successfully used to measure the degree of order of the myosin heads along the thick filament backbone (40, 41). An x-ray comparison between βWT and R1500P myofiber bundles showed that the peak intensity of the first and the second myosin meridional reflections at 14.5 and 7.2 nm (MM1 and MM2, respectively) was reduced in the mutant samples (Figure 4C); while MM1 arises from the axial spacing of the myosin heads on the thick filament, MM2 arises from thick the filament backbone periodicity. The observed intensity reduction of the 2 peaks was not accompanied by any change in their reciprocal spacing. Since the intensity of MM1 and MM2 reflections appeared to be positively correlated with the number of ordered myosin heads helically arranged along the thick filaments, these results are consistent with the idea that more myosin molecules adopted a DRX conformation and suggest the presence of a distortion of the periodic structure of the rod introduced by the proline mutation.

### Muscle fitness is decreased in R1500P mice.

**Figure 5:**
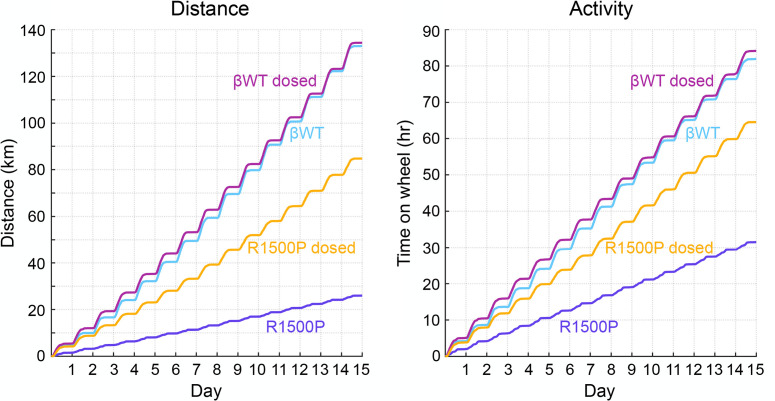
The compromised running performance of the transgenic R1500P mice is partially rescued by oral treatment with the myosin modulator MYK-581. Mouse locomotion analysis was recorded over 15 days of voluntary wheel running. Actograms of distance and activity are shown. Four-month-old male mice were acclimated to the running wheel cages for 4 days before data collection was commenced. Kilometers run (distance) and hours spent on the wheel (activity) are plotted as a function of the total experimental time measured in days. Dosed groups received MYK-581 (10 mg/kg) via chow starting on the first day of acclimatization. βWT mice, *n* = 7; βWT dosed mice, R1500P mice, and R1500P dosed mice, *n* = 10/group.

**Figure 6:**
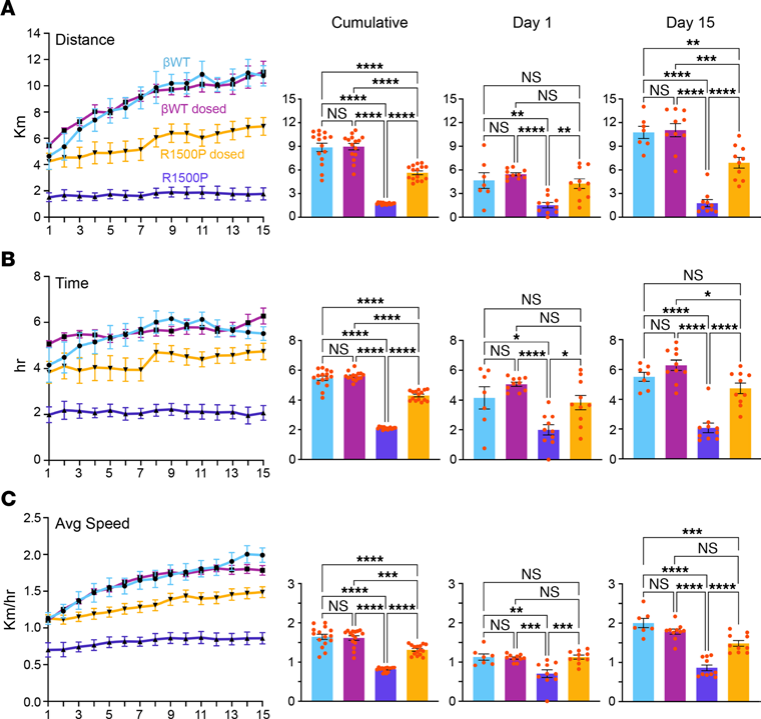
MYK-581 significantly ameliorates the running performance of transgenic R1500P mice. (**A**) Daily average distance (Km) covered by each group of 4-month-old male mice over 15 days is shown on the left; statistical analysis of the cumulative distance as well as the comparison between day 1 and day 15 is shown on the right of the panel. (**B** and **C**) Group activity (h) and average (Avg) speed (Km/h). βWT mice, *n* = 7; βWT dosed mice, R1500P mice, and R1500P dosed mice, *n* = 10/group. All data are expressed as the mean ± SEM. *P < 0.05, **P < 0.01, ***P < 0.001, and ****P < 0.0001, by 2-way ANOVA (genotype × drug) with Bonferroni’s multiple-comparison post hoc test.

Next, mice underwent a voluntary wheel-running test to assess the effects of the R1500P mutations on muscle endurance and capacity. In view of the higher percentage of myosin heads shifted in the DRX configuration found in the MYH7-mutant myofibrils, we also examined the in vivo therapeutic efficacy of MYK-581, after daily administration via dosed chow for the duration of the running training. Distance and activity actograms of the βWT and R1500P mouse groups, monitored over a 15-day period, showed no apparent disruption of the circadian resting (rho [ρ]) or active (alpha [α]) phases in any of the groups (Figure 5 and Supplemental Figure 8). Conversely, the drop in total distance and activity (~80.5 and 62%, respectively) observed for mice in the R1500P group substantiated the presence of a distal myopathy in the mutant strain. Notably, treatment with MYK-581 ameliorated the running phenotype of the R1500P animals by increasing both the distance and activity of the R1500P dosed group by approximately 30% and 48%, respectively, without apparent changes in the performance of the βWT control dosed group. Higher-granularity analysis of the daily running data (Figure 6, A–C) revealed that the distance, activity, and average speed of the βWT group increased logarithmically through training, as described for WT mice (42). In contrast, mice in the R1500P group showed no improvement in the 3 running parameters over time. Notwithstanding, administration of the myosin modulator partially rescued the distance and speed phenotype of mice in the R1500P group (Figure 6, A and C, R1500P dosed), while mainly flattening the rate of activity change for both βWT and R1500P groups (Figure 6B, βWT dosed, R1500P dosed). Cumulative statistical analysis of daily distance, activity, and speed showed a significant increase in the performance of the R1500P dosed mice over the R1500P group (3.2-, 2.0-, and 1.7-fold, respectively), remaining, however, significantly lower than the performance of mice in the βWT group (1.5-, 1.2-, and 1.3-fold, respectively) (Figure 6, cumulative panels, and Supplemental Table 1). Group comparison statistics at day 1 of recorded exercise (Figure 6, A–C; day 1 panels), which corresponds to day 5 of MYK-581 therapy initiated during the cage acclimatization period, showed that the running proficiency of the R1500P dosed group was already significantly better than that of the R1500P group and equal to both βWT groups. However, statistical analysis of mouse running parameters at day 15 confirmed that after this early and striking improvement, the R1500P dosed group did not respond to the training as well as the βWT groups did (Figure 6, A–C; day 15 panels).

**Figure 7:**
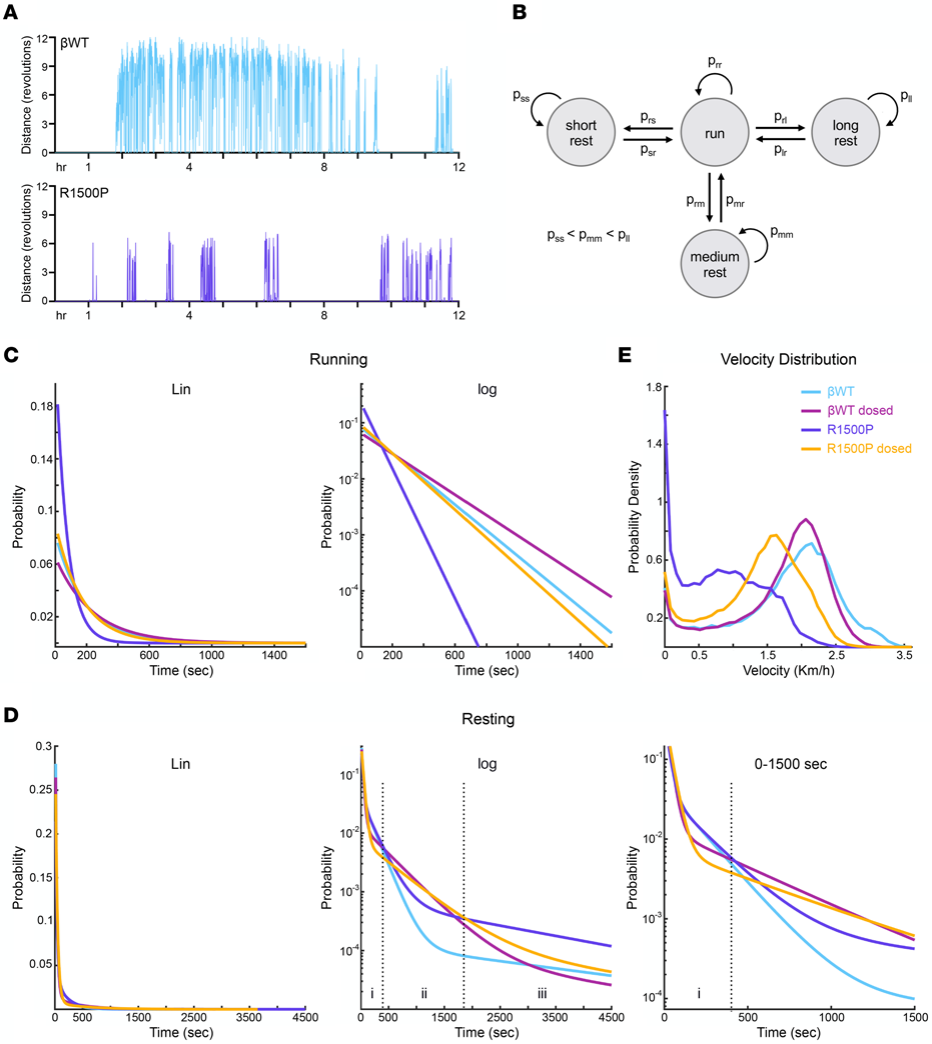
Markov model of mouse activity shows hidden running patterns. (**A**) Running wheel activity of 2 representative 4-month-old βWT and R1500P male mice recorded during the circadian α phase. (**B**) Diagram of the discrete-time Markov chain used for modeling mouse running bouts and rests. The model transitions between 1 running and 3 resting states. The condition p_ss_ < p_mm_ < p_ll_ guarantees that leaving the short, medium, and long rest states is increasingly difficult. (**C**) PMFs associated with the Markov model, giving the probability of the duration of every single run and rest. PMFs are shown with a linear (Lin) and a logarithmic (log) *y* axis. (**D**) PMFs associated with the Markov model describing the duration of a single resting bout. PMFs are shown with a linear and a logarithmic *y* axis. The last panel on the right reports the first 1,500 seconds of the log plot. Time windows of interest are labeled i–iii. (**E**) Probability density plot reporting the velocity distributions of the groups as determined experimentally. βWT mice, *n* = 7; βWT dosed mice, R1500P mice, and R1500P dosed mice, *n* = 10/group.

Close inspection of the running patterns revealed that the 12-hour activity profile of the R1500P group, characterized by sporadic running bouts separated by long resting periods, was prominently different from the one recorded for the βWT group (Figure 7A). To explore this finding in greater detail, we modeled mouse activity during the active circadian phase α using a discrete time Markov chain (43). Specifically, we introduced a Markov chain that transitions between the 4 states “run,” “short rest,” “medium rest,” and “long rest.” We computed the model transition probabilities to maximize the likelihood of obtaining the experimental data that comprise running and resting sequences recorded over 15-second periods (Figure 7B and Supplemental Figures 9 and 10). The simple structure of the Markov model allowed us to derive closed-form expressions for various quantities including average running and resting times and the probability mass functions (PMFs) describing the duration of runs and rests.

The average run and rest times demonstrated that the administration of MYK-581 had a curative effect on mouse muscle performance by increasing the mean run time of the R1500P dosed group to approximately 3 minutes, while decreasing its mean rest time to approximately 5 minutes, with the R1500P group at approximately 1.3 and 6.9 minutes, respectively (Table 1). In spite of the fact that MYK-581 lowers the DRX population of myosin molecules competent for actin binding after thin filament activation, the mean run of the βWT dosed group was 1.2-fold higher than that of the βWT control group; however, this unexpected rise was precisely counterbalanced by a 1.2-fold longer mean rest time (Table 1). PMFs generated by the Markov model provided, however, a more detailed description of running performance than did a simple mean and SD; moreover, unlike the experimental data, they were also free of noise. The PMF for a single running bout (Figure 7C, linear and logarithmic graphs) showed that the R1500P group had a higher probability of executing short runs (0–150 seconds) and a significantly lower probability of performing long runs (>150 seconds) in comparison with all the remaining groups. Nevertheless, MYK-581 administration shifted the running curve of the R1500P dosed group rightward near the curve for the βWT control group, bringing its performance close to βWT levels across all the run durations. The plots also reveal the positive effect of MYK-581 on the βWT dosed group, predicting an increased probability of the mice executing long runs (e.g., the probability of a 25-minute run being approximately 5 times greater than that for the βWT mice).

The PMF for a single resting bout (Figure 7D, linear and logarithmic graphs) showed 3 prominent time windows of interest: region A (~0–400 seconds), where the βWT and R1500P groups had a decreased probability of resting compared with their dosed counterparts; region B (~400–1,900 seconds), where the opposite occurred and both βWT and R1500P had an increased probability of resting; and region C (~1,900–4,500 second), where the R1500P group showed the highest probability of resting for long periods. Last, using experimentally measured velocities, we approximated the probability density function for each group velocity (Figure 7E). This complementary analysis validated the therapeutic effects of MYK-581 in restoring mutant muscle performance, while positioning the βWT as the fastest group in the 3–3.5 Km/h range.

To assess cardiac function, potentially affected by the inhibitory effect of MYK-581 on cardiac myosin, we next performed an echocardiogram on mice in the 2 dosed groups. This analysis showed a significant decrease in several parameters, such as fractional shortening and ejection fraction for the βWT group, but only a nonsignificant trend was observed for the R1500P group (Supplemental Figure 11). Altogether the muscle strength and fitness data presented here, augmented by the Markov model analysis, confirmed both the negative effect of the R1500P mutation on muscle activity, as well as the effectiveness of our therapeutic intervention with a myosin modulator.

## Discussion

Approximately 60% of the MPD1 missense mutations are proline substitutions distributed in each of the 7 positions of the MYH7 coiled-coil heptad repeat (Supplemental Figure 12). It is well established that proline residues introduce a sharp regional bending of the helix axis due to their inability to take part in proper hydrogen bonding and to the cyclic structure of the proline side chain clashing with the α-helix (44). A limited but exhaustive analysis of the crystal structure of trimeric and dimeric coiled-coils with internal prolines suggests that their accommodation can occur via local over- or underwinding of the α-helix, transitioning to the tighter 3_10_-helix or the looser α-helix (45). Although a proline-induced 3_10_-helix could occur in any position of the heptad repeat, a α-helix could form only in the outer positions *b*, *c*, and *f* (the R1500P mutation being in the *f* position). Furthermore, molecular dynamics simulations of the pathogenic MYH7 mutations R1588P, L1591P, and A1603P, (*b*, *c*, and *e* positions, respectively) show a substantial bend in the rod coiled-coil (15, 46). Since the thick filament contains approximately 300 myosin molecules, the regional distortion/flexibility caused by proline kinks may become additively damaging for the overall stability of the thick filament. Consistently, paracrystals formed by the R1500P and L1793P myosin mutants appear more accessible to trypsin proteolysis (47, 48). Given that, theoretically, a distance of 96 nm separates the myosin head and the R1500P rod mutation (0.1485 nm/residue) and that this rather long distance should insulate the head from an intramolecular propagation of the mutation effects, we propose that proline mutations structurally warp the staggered array of adjacent myosin rods in the thick filament. Consequently, both the sarcomeric actin-myosin and myosin-myosin interfaces become misaligned, affecting both myosin cross-bridge cycling and the SRX/DRX ratio. Accordingly, we found that the rate of actin-myosin detachment in isolated mutant myofibrils (49, 50) was almost 2-fold faster (1.95 times), while its duration (*t*_REL_) was significantly shorter (0.85 times). Muscle tension cost corresponds to the ratio between ATPase activity and force, and faster cross-bridge ingress into non-force-generating states is predicted to decrease maximal force production; higher energetic costs are therefore required to generate and maintain adequate muscle tension (51). Our data also showed a significant shift in the proportion of heads in DRX conformation, which had a 10-fold higher ATP turnover rate, enabled cross-bridge formation, and enhanced muscle contractility. Moreover, during the submission of this manuscript, Carrington et al. reported that a number of myosin mutations localized in the LMM region of the rod and causing human skeletal myopathy also increase the number of myosin heads in the DRX state (52). Several observations explain why myosin rod mutations can affect head energetics. During relaxation, the 2 heads of myosin can engage and form the interacting heads motif (IHM). In this structural conformation, which is distinctive of molecules in SRX, the IHM becomes sequestered by the proximal part of the myosin rod called subfragment 2 (S2). This reduces ATP turnover by trapping and preventing the release of the ATP hydrolysis products (53). Importantly, the comparison of molecules in the SRX state measured in single- and double-headed constructs (~10%–20% and ~25%–30%, respectively) and muscle fibers (~60%–75%) indicate that when folded in the sarcomere, IHMs establish a distinctive network of anchoring intermolecular interactions among themselves, neighboring myosin rods of the filament backbone, and other sarcomeric proteins (54, 55). It is relevant to note that a detailed and comparative analysis of the tarantula IHM homology model identified an intermolecular interaction between the blocked head with neighboring subfilaments (56). According to the mechanosensing model, an increase in the external muscle load of as little as approximately 1% activates vertebrate thick filaments by inducing mechanical stress in the filament backbone that unfolds the helical packing of the IHMs (57). Likewise, in view of the intensity decrease of the R1500P meridional reflection MM2, which arises from the filament backbone, we propose that the global distortion of the filaments induced by proline rod mutations mechanically enhances myosin off-to-on transition. Thus, the structural integrity observed in sarcomeres of our transgenic mice and in patient does not necessarily equate with functional integrity. Cryo-EM reconstruction of human and mouse cardiac thick filaments has been recently reported (58–60). We have mapped the R1500P mutation on the 3D reconstruction of the thick filament C-zone (PDB: 8G4L) and found that in each of the 43 nm sarcomeric repeats, the proline substitution was periodically located in close proximity to one of the globular domains of each of the 6 titin molecules, as well as all the third myosin heads (~1.5 and ~2.5 nm, respectively). Thus, in addition to a general effect of proline mutations on the thick/thin filament register, the proline-induced distortion of the coiled-coil could increase the number of myosin heads in the DRX state by destabilizing the intermolecular interactions occurring between a subpopulation of IHMs and the other components of the thick filament. There are only a few reports investigating the effect of pathogenic β-myosin mutations on myofilament calcium sensitivity. For example, it has been reported that ventricular papillary muscles transgenically expressing the HCM mutation R403Q show increased calcium sensitivity (61). Direct effects of calcium on increasing the myosin population in the DRX state by relieving head-backbone interactions that stabilize the SRX state have recently been proposed (62). Thus, we believe that mutations increasing the DRX/SRX ratio induce increased calcium sensitivity by virtue of the higher number of heads prepared to interact with actin before calcium transients. As a result, even though the number of myosin heads activated at a low calcium concentration is proportionately small, it will likely be sufficient to cross the threshold of thick filament activation. Accordingly, we predict increased calcium sensitivity only for the mice expressing the R1500P mutant myosin.

Our broad analysis of muscle mechanical behavior and performance suggests that incorporation of the R1500P mutation into the sarcomere causes hypercontractility, muscle fatigue (temporary decrease in the ability to perform a physical task), and weakness (decrease in muscle strength), the latter being a clinical hallmark of MPD1. The significant rescue of the R1500P running phenotype after pharmacological rebalancing of the sarcomeric SRX/DRX content confirms the importance of myosin conformational states in causing muscle disease, as proposed for patients with HCM and left ventricle outflow tract obstruction (63). Moreover, after 4 days of MYK-581 therapy initiated during the period of cage acclimatization, the running capacity of the R1500P group was statistically equivalent to that of the βWT group, indicating a fast reversal of the myopathy and suggesting inefficient ATP usage and/or energy depletion. Conversely, the incomplete performance recovery after 14 days of training could be ascribed to the faster cross-bridge detachment measured in the R1500P myofibrils, which MYK-581 does not modulate. Taken together, these data suggest both hyper- and hypocontractile R1500P phenotypes: while hypercontractility prevailed at baseline and was corrected by MYK-581 treatment, hypocontractility predominated after drug administration and DRX rebalancing. In our exercise paradigm, we observed no improvement or deterioration in the running performance of the R1500P group at the end of the exercise training period. This was not unexpected, since the beneficial effects of strength and aerobic exercise training on different myopathies remain somewhat uncertain (64). Testing the efficacy of MYK 581 in transgenic male mice only could be regarded as a potential limitation of our therapeutic approach. However, a recent study has shown that mavacamten treatment produced comparable positive responses in men and women affected by symptomatic obstructive HCM (65).

The examination of running performance based on our Markov model yielded several insights and subtle differences in the activity patterns of βWT and mutant mice. In particular, PMFs for running/rest duration revealed that the MYK-581–dosed βWT and R1500P groups executed long runs more frequently, had fewer short rests, and had more medium rests than did the corresponding untreated βWT and R1500P groups. Importantly, these running features were not highlighted by routine cumulative analyses of distance, activity, or velocity. Although the clinical benefits of the myosin inhibitor were consistent with the DRX phenotype, it is puzzling why the performance of the βWT group was not negatively affected by the drug, considering the high expression of β-myosin in our transgenic animals. However, by reducing the rate of phosphate release of β-myosin, MYK-581 could reduce energy expenditure and increase energy supply during exercise (66). Moreover, the relative behavior of the curves observed in regions A and B of the resting PMF suggests that the higher running activity predicted for the dosed groups elicited compensatory intervals of muscle recovery. Our model accurately simulated mouse running and resting behavior, as evidenced by the good agreement with experimental data and, more generally, with the running and resting PMFs (Supplemental Figure 13 and Supplemental Table 2). The Markov model described here can be broadly applied to quantify running performance in disease and test the efficacy of drugs. Our approach may also prove useful for extracting detailed running and resting statistics when only a limited quantity of experimental data is available.

In summary, the general model proposed here for proline rod mutations causing skeletal myopathies is based on the following sequence of events: (a) formation of a local kinking of the coiled-coil caused by unique proline structural properties; (b)spreading of the distortion along the sarcomere by myosin staggered assembly; and (c) a shift in the SRX/DRX ratio with a subsequent increase in the muscle metabolic rate as reported for a subset of HCM myosin mutations located in the motor domain (67–70). The restoration of good metrics of muscle physical function and mobility achieved by reprogramming myosin enzymatic activity via oral administration of a small-molecule modulator raises hope for patients with MPD1 and related MYH7 distal myopathies.

## Methods

### Sex as a biological variable.

Our study examined male mice only because male animals showed less phenotypical variability.

### Generation of WT and R1500P MYH7–transgenic mice by standard transgene approach.

The generation of transgenic mice was performed at the University of Colorado, Boulder, Transgenic Mouse Facility. cDNA synthesis from mouse soleus RNA and PCR reactions using the primers KozATGβ, forward: CCCACCATGGCGGATGCAGAGATGGCTG (adding the Kozak consensus sequence) and BetaStop, reverse: CTACTCCTCATTCAGGCCCTTGGCAC (including the stop codon) were used to isolate the mouse *MYH7* coding sequence. The resulting 5,814 bp PCR product was then subcloned into a BlueScript vector linearized with EcoRV, downstream of a 4,800 bp region of the mouse MCK gene (19). Subsequently, the Myc tag VEQKLISEEDL was inserted at the C terminus of the MYH7 gene by inverse PCR using the primers Myc, forward: TCTGAAGAGGACTTGTGAATCGAATTCCTGCAGCCCAGCTTG and Myc, reverse: AATGAGCTTTTGCTCCACGGCACCAATGTCCCGGCTCTTGGCs; the Myc tag replaced and extended the end of the MYH7 sequence KGLNEE. The R1500P mutation was introduced into the WT construct by inverse PCR using the primers R1500P, forward: CCGGAGAACAAGAACCTCCAG and R1500P, reverse: CTTGAAGGTCTCTAGGTGCTC. The WT and mutant constructs were then digested with NotI and PvuI, and the purified 11,608 bp linear fragments were used for microinjection into Friend virus B NIH Jackson (FVB/NJ) donor mice oocytes that were subsequently implanted into CD1 foster mice. The progeny was genotyped from tail clips with primers specific for the C-terminal portion of MYH7 and the Myc tags Gtype, forward: GCAGAGGAGAAGGCCAAGAAGG and Gtype, reverse: CTCTTCAGAAATGAGCTTTTGCTC, respectively. We obtained 15 βWT and 5 βR1500P founders that were bred with JaxC57Bl6 mice (The Jackson Laboratory).

### Human muscle biopsy specimens.

Human muscle biopsy specimens were obtained from patients diagnosed with distal myopathies and who had specific heterozygous *MYH7* mutations and from age-matched controls with no history of neuromuscular diseases. While the R15060P mutation was previously described (71), the L14567P mutation is novel and was identified in a 35-year-old-female who was first noted to have difficulties with stairs and shoulder strength in her twenties. In retrospect, she was never very athletic in childhood, and she had always been a slow runner. Her early motor development was otherwise unremarkable. Over the years, her weakness gradually progressed, predominantly affecting the distal lower extremities and neck flexion. Her family history is significant for a mother with similar symptoms. Neuromuscular examination at age 35 years revealed distal (Medical Research Council [MRC] scale, 4/5) greater than proximal (MRC, 5/5) weakness with axial involvement (neck flexion, 2/5). The patient’s muscle mass was reduced throughout. Her reflexes were 1+ in the arms and legs and absent in the ankles. Spinal examination revealed striking spinal rigidity with prominent paraspinal muscles. There was left > right scapular winging. The patient had mild contractures of the heel cords bilaterally and of the finger flexors bilaterally. She had more notable contractures of the neck musculature, with limited lateral rotation of the neck and limited neck flexion. There was no significant joint hypermobility. The patient’s gait was described as normal, with poor heel strike. She was able to arise from a supine to a sitting position by rolling to the side and pushing off her elbow to a sitting position. Creatine kinase levels had been mildly elevated, ranging from 179–309 units/L. Electrodiagnostic studies were suggestive of a chronic myopathic process affecting distal and proximal muscles. A muscle ultrasound was suggestive of myopathy with more striking involvement of distal muscles in the leg (anterior compartment > posterior compartment); hamstring > quadriceps; and paraspinal muscles. A muscle biopsy of the right thigh at age 34 years revealed mild myopathic abnormalities including fiber size variability, internally placed nuclei, type 2 predominance, and rare vacuolated fibers. A few myofibers showed mild and focal irregularity in interfibrillar organization. Immunohistochemical staining for dystrophin, α-sarcoglycan, merosin, dysferlin, caveolin 3, α-dystroglycan, and utrophin was normal. A desmin immunostain revealed well-organized sarcomeric patterns in most fibers; however, mild and focal sarcoplasmic irregularities were also sporadically observed. Genetic testing revealed a c.4400T>G; p.Leu1467Pro *MYH7* variant that was inherited from the patient’s affected mother.

### Muscle isolation and quantification.

Mouse hind limb muscles, including gastrocnemius, soleus, EDL, and TA, were removed following standard protocol (72), cleaned of extraneous tissue, blotted, and cryopreserved for downstream assessments. TA muscles were weighed using an analytical scale for muscle weight assessments.

### Muscle lysate preparation.

Frozen muscle tissues were pulverized while dry using a BioPulverizer (MidSci). The pulverized muscle powder was then suspended in a 5% SDS, 0.75% sodium deoxycholate, 50 mM Tris-HCl (pH 8.0) buffer and heated at 95°C for 10 minutes. The muscle lysate was then centrifuged at 12,000*g* for 10 minutes, and the supernatant was collected for downstream mass spectrometric analysis. The protein concentration was determined by bicinchoninic acid (BCA) assay.

### Mass spectrometry sample preparation.

Mouse muscle lysates were denatured in 5% (w/v) SDS, 50 mM Tris-HCl, pH 8.5, 10 mM tris(2-carboxyethylphosphine) (TCEP), and 40 mM chloroacetamide by boiling at 95°C for 10 minutes. Each sample was digested using the SP3 method (73). Carboxylate-functionalized speedbeads (GE HealthCare Life Sciences) were added to the lysates. Addition of acetonitrile to 80% (v/v) caused the proteins to bind to the beads. The beads were washed twice with 80% (v/v) ethanol and twice with 100% acetonitrile. Proteins were digested with Lys-C/Trypsin (Promega) overnight, rotating at 37°C. Speedbeads were collected by centrifugation and then placed on a magnet to remove the digested peptides. The peptides were then desalted using an Oasis HLB cartridge (Waters), according to the manufacturer’s instructions, and dried in a SpeedVac (Thermo Fisher Scientific).

### Mass spectrometric analysis.

Samples were suspended in 3% (v/v) acetonitrile/0.1% (v/v) trifluoroacetic acid, and 1 μg tryptic peptides was directly injected onto a C18 1.7 μm, 130 Å, 75 μm × 250 mm M-class column (Waters) using a Thermo Ultimate 3000 RSLCnano UPLC (Thermo Fisher Scientific). Peptides were eluted at 300 nL/min using a gradient from 2%–20% acetonitrile over 100 minutes into a Q-Exactive HF-X mass spectrometer (Thermo Fisher Scientific). Precursor mass spectra (MS1) were acquired at a resolution of 120,000 from 350–1550 *m/z* with an automatic gain control (AGC) target of 3E6 and a maximum injection time of 50 milliseconds. The precursor peptide ion isolation width for MS2 fragment scans was 1.4 *m/z*, and the top 12 most intense ions were sequenced. All MS2 spectra were acquired at a resolution of 15,000 with higher energy collision dissociation (HCD) at 27% normalized collision energy. An AGC target of 1E5 and a 100-millisecond maximum injection time were used. Raw files were searched against the Uniprot *Mus musculus* databases UP000000589 using MaxQuant, version 2.0.3.0, with cysteine carbamidomethylation as a fixed modification. Methionine oxidation and protein N-terminal acetylation were searched as variable modifications. All peptides and proteins were thresholded at a 1% FDR.

### Western blotting.

Protein lysates were prepared by homogenizing hind limb muscle tissue in myosin extraction buffer (0.3 M NaCl, 0.1 M NaH_2_PO_4_, 0.05 M Na_2_HPO_4_, 0.001 M MgCl_2_.6H_2_O, 0.01 M EDTA) following standard procedures. The antibodies used were against Myc tag (9B11, Cell Signaling Technology; 1:10,000 dilution) and α-sarcomeric actin (A2172, MilliporeSigma, 1:2,000 dilution). All blots were imaged using the ImageQuant LAS 4000 (GE Healthcare Bio-Sciences) system and analyzed with ImageQuant and/or ImageJ (NIH) software.

### Succinate dehydrogenase staining.

TA muscles were snap-frozen in isopentane/liquid N_2_, cryosectioned, and stained for enzymatic activity analysis using standard procedures. The stained fibers were counted, and their percentage of total number of fibers was calculated (150–200 total fibers/image, 3 images/mouse, 2 mice/genotype). The CSA was determined using ImageJ.

### TEM.

TEM studies were carried out as described in a previously published procedure (74).

### Myofibril immunostaining.

Myofibril preparation and immunostaining were performed as previously described (75) using the Myc tag antibody 9B11 (Cell Signaling Technology; 1:250 dilution).

### Ex vivo contractility assay.

Mice were euthanized according to NIH guidelines and IACUC institutional animal protocols. The EDL was carefully dissected in total from the ligamentary attachment at the lateral condyle of the tibia to the insertion region. The muscle was transferred to a dish containing ice-cold isotonic physiologic salt solution (Tyrode’s buffer: 118 mM NaCl, 4 mM KCl, 1.2 mM MgSO_4_, 25 mM NaHCO_3_, 1.2 mM NaH_2_PO_4_, 10 mM glucose, and 2.5 mM CaCl_2_) bubbled with 95% O_2_/5% CO_2_ to maintain a pH of 7.4. The soleus was identified after removing the gastrocnemius muscle and was removed by cutting the ligaments connecting to the proximal half of the posterior tibia to the insertion, where the calcaneal tendon was cut, and the muscle was placed in ice-cold Tyrode’s buffer. Muscles were mounted vertically in individual tissue bath chambers and maintained at 37°C. Muscles were stretched, and optimal length was set for each muscle. Stimulatory trains of varying frequency (1–100 Hz) were used to generate force-frequency curves. Tetanic force was achieved in all muscles using 100 Hz.

### Myofibril isolation.

Myofibrils were isolated from flash-frozen soleus and TA muscles as described previously (76, 77). A small section of muscle was cut into thin slices and bathed in 0.05% Triton X-100 in Linke’s solution (132 mM NaCl, 5 mM KCl, 1 mM MgCl_2_, 10 mM Tris, 5 mM EGTA, 1 mM NaN_3_, pH 7.1) with a protease inhibitor cocktail (10 μM leupeptin, 5 μM pepstatin, 200 μM phenyl-methylsuphonylfluoride, 10 μM E64, 500 μM NaN_3_, 2 mM dithioerythritol) overnight at 4°C. Skinned tissue was washed 3 times in rigor solution (50 mM Tris, 100 mM KCl, 2 mM MgCl_2_, 1 mM EGTA, pH 7.0) and resuspended in bath solution with protease inhibitors (pCa 9.0; 100 mM Na_2_EGTA; 1 M potassium propionate; 100 mM Na_2_SO_4_; 1 M MOPS; 1 M MgCl_2_; 6.7 mM ATP; and 1 mM creatine phosphate; pH 7.0). The tissue was then homogenized at medium speed 3 times for 10 seconds each.

### Myofibril mechanics.

Myofibril mechanical parameters were measured as described previously (77–79). Myofibrils were placed on a glass coverslip in relaxing solution at 15°C, and then a small bundle of myofibrils was mounted onto 2 microtools. One microtool was attached to a motor that produces rapid length changes (Mad City Labs), and the second microtool was a calibrated, cantilevered force probe (5.8 μm/μN; frequency response 2–5 KHz). Myofibril length was set at 5%–10% above slack length and average sarcomere length, and myofibril diameters were measured using ImageJ. Mounted myofibrils were activated and relaxed by rapidly translating the interface between 2 streams of solutions of pCa 4.5 and pCa 9.0 (79, 80). Data were collected and analyzed using customized LabView software. The measured mechanical and kinetics parameters were defined as follows: (a) resting tension (mN/mm^2^), myofibril basal tension in the fully relaxing condition; (b) maximal tension (mN/mm^2^), maximal tension generated at full calcium activation (pCa 4.5); (c) the rate constant of tension development following maximal calcium activation (*k*_ACT_); (d) the rate constant of tension redevelopment following a release-restretch applied to the activated myofibril (*k*_TR_) (51); (e) the rate constant of early slow force decline (*k*_REL,_ _LIN_) — slope of the linear regression normalized to the amplitude of relaxation transient, duration of early slow force decline — measured from the onset of solution change to the beginning of the exponential force decay, and (f) the rate constant of the final exponential phase of force decline (*k*_REL,_ _EXP_).

### Mant-ATP chase experiments.

Single-membrane permeabilized muscle fibers were isolated in the relaxing buffer. Their ends were individually clamped to half-split copper meshes designed for electron microscopy (SPI G100 2010C-XA, width, 3 mm), which had been glued to glass slides (Academy, 26 × 76 mm, thickness 1.00–1.20 mm). Coverslips were then attached to the top using double-sided tape to create flow chambers (Menzel-Gläser, 22 × 22 mm, thickness 0.13–0.16 mm). The mean sarcomere length was set to 2.2 μm.

At 25°C, similar to previous protocols (38), each myofiber was first incubated for 5 minutes with a rigor buffer (composition: 120 mM K acetate, 5 mM Mg acetate, 2.5 mM K_2_HPO_4_, 2.5 mM KH_2_PO_4_, 50 mM MOPS, 2 mM DTT, at pH 6.8). A solution containing the rigor buffer with 250 μM Mant-ATP was then flushed and kept in the chamber for 5 minutes. At the end of this step, another solution made of the rigor buffer with 4 mM ATP was added with simultaneous acquisition of the Mant-ATP chase. For fluorescence acquisition, a Zeiss Axio Scope A1 microscope was used with a Plan-Apochromat ×20/0.8 objective and a Zeiss AxioCam ICm 1 camera. Frames were acquired every 5 seconds with a 20-millisecond acquisition/exposure time using a DAPI filter set, and images were acquired for 5 minutes. Similar to protocols previously described for analysis (38), three regions of each individual muscle fiber were sampled for fluorescence decay using the region-of-interest (ROI) manager in ImageJ. The mean background fluorescence intensity was subtracted from the average of the fiber fluorescence intensity. Each time point was then normalized by the fluorescence intensity of the final Mant-ATP image before washout (*T* = 0). These data were then fit to an unconstrained double exponential decay using Sigmaplot: normalized fluorescence = 1 − *P1* (1−exp^(−t/T1)^) − *P2* (1−exp^(*−t/T2*)^), where *P1* is the amplitude of the initial rapid decay approximating the DRX state, with *T1* as the time constant for this decay, and *P2* is the slower second decay approximating the proportion of myosin heads in the SRX state with its associated time constant *T2*. To obtain DRX and SRX values, *P1* and *P2* were adjusted for the level of nonspecific binding found to be 40% in skeletal muscle (37).

### x-ray diffraction recordings and analyses.

Thin muscle bundles were placed in a plastic dish containing a relaxing solution (composition: 4 mM Mg-ATP, 1 mM free Mg^2+^, 20 mM imidazole, 7 mM EGTA, 14.5 mM creatine phosphate, 324 U/mL creatine phosphokinase, 1,000 U/mL catalase, and KCl to adjust the ionic strength to 180 mM and pH to 7.0). They were then transferred to the specimen chamber, filled with the relaxing buffer. The ends of these thin muscle bundles were then clamped at a resting sarcomere length (approximately 2.2 μm). Subsequently, x-ray diffraction patterns were recorded at 15°C. For each preparation, approximately 20–30 diffraction patterns were recorded at the BL40XU beamline of SPring-8 and analyzed as described previously (81). To minimize radiation damage, the exposure time was kept low (2 seconds), and the specimen chamber was moved by 100 μm after each exposure. Following the x-ray recordings, background scattering was subtracted, and the major MM reflection intensities/spacing were determined as described previously (82). The equatorial intensities are not shown in our analysis for the following reasons: (a) there was a slight misalignment due to the use of bundles rather than single fibers, which suggests heterogeneous sarcomere organization; consequently, some of the 1,1 reflections were missing and not analyzed in the corresponding acquired images; and (b) the 1,0 reflection was sometimes unusually wide or very faint. To avoid any nonphysiological artifact when assessing the 1,1/1,0 ratio (equals overestimation of this ratio), we did not report the data. Given the above, our data were focused only on strong and relevant reflections.

### Four-limb hanging test.

Male mice were placed in the center of a wire mesh screen, a timer was started, and the screen was rotated to an inverted position with the mouse’s head declining first. The screen was held above a padded surface. Either the time when the mouse fell was noted ,or the mouse was removed when the criterion time of 60 seconds was reached.

### Grip strength.

Forelimb grip strength was measured with a grip strength meter. The mice were first acclimatized to the apparatus for approximately 5 minutes. Individual mice were then allowed to grab the bar while being held from the tip of their tail. The mouse was gently pulled away from the grip bar. When the mouse could no longer grasp the bar, the reading was recorded. The protocol was repeated 5 times with at least a 30-second rest between trials. The highest 3 values were averaged to obtain the absolute grip strength.

### Voluntary wheel running.

βWT and R1500P male mice were individually housed in cages containing a running wheel with a magnetic sensor connected to an Arduino microcontroller interfaced with a PC computer for data storage. Mice were acclimatized to the running wheel cages for 4 days before data collection was commenced. Running activity (wheel revolutions) was recorded over 15-second intervals. Mice had unlimited access to the running wheels for the duration of the experiments. The myosin modulator MYK-581 was administered ad libitum in the mouse chow (Purina, 5001) at 10 mg/kg (incorporation by Research Diets Inc.) since the first day of acclimatization.

### Mouse echography.

Mouse echography was carried out as previously described (83).

### Markov model.

The proposed Markov model for modeling mouse activity is characterized by the 10 transition probabilities *p_rr_*, *p_rs_*, *p_rm_*, *p_rl_*, *p_ss_*, *p_mm_*, *p*_μ_, *p_sr_*, *p_mr_*, and *p_lr_*.

These transition probabilities can be conveniently represented using the transition matrix


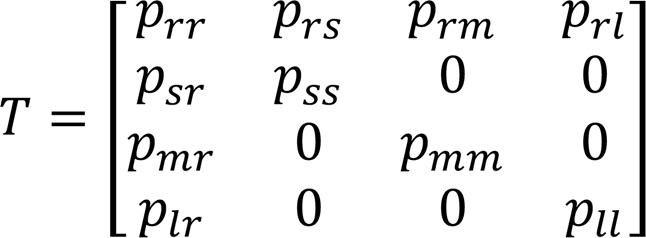
 Equation 1

Since the sum of the transition probabilities corresponding to each state must add up to one, 3 of these parameters are dependent, leaving a total of 6 free parameters. In the discussion that follows, we chose to keep *p_rs_*, *p_rm_*, *p_rl_*, *p_ss_*, *p_mm_*, and *p*_μ_*,* free and take

*p_rr_* = 1 – *p_rs_* – *p_rm_* – *p_rl_*, *p_sr_* = 1 – *p_ss_,* *p_mr_* = 1 – *p_mm_,* *p_lr_* = 1 – *p*_μ_

The transition probabilities that maximize the likelihood of observing the experimental data for each mouse group, rounded to the ten thousandths digit, are


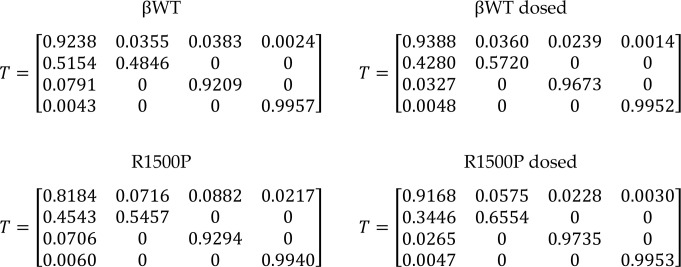


We numerically computed these transition probabilities by applying a gradient descent to minimize the negative log likelihood. For a given activity sequence, the likelihood function is


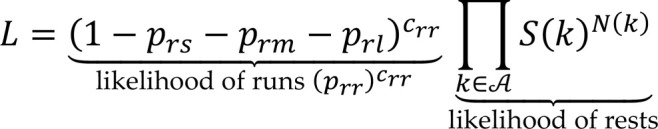
 Equation 2

where *c_rr_* is the number of transitions from the state “run” back to “run” state, 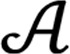 is the set of unique resting lengths, N(*k*) represents the number of times a resting length k appeared in the activity sequence, and S(*k*) is the likelihood of a rest of length *k* given by


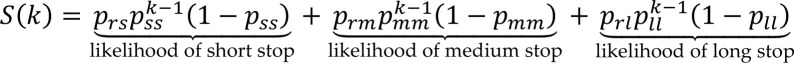
 Equation 3

When we observe a rest period of length *k* in an experimentally obtained activity sequence, we cannot determine whether it corresponds to a short, medium, or long rest. Therefore, the likelihood S(*k*) weighs each of these 3 possibilities accordingly.

### Statistics.

Data are presented as the mean ± SEM. Differences between groups were evaluated for statistical significance using Student’s 2-tailed *t* test (2 groups) or 1- or 2-way ANOVA (more than 2 groups) followed by Bonferroni’s post hoc test for pairwise comparisons. *P* values of less than 0.05 were considered significant unless otherwise noted.

### Study approval.

All animal experiments were performed using protocols approved by the IACUC of the University of Colorado (protocol no. 235116FEB2019). Animals were housed under standard conditions in a partial barrier facility and received ad libitum access to water and chow. For sample collection, animals were sedated using 1%–4% inhaled isoﬂurane and sacrificed by cervical dislocation. All data shown are from male mice. The x-ray experiments were performed under approval of the SPring-8 Proposal Review Committee (Japan, approval no. 2020A1050).

Written informed consent was obtained from all participants or their legal representative. The National Research Ethics Service (NRES) Committee North East (United Kingdom) approved all the experiments (REC 13/NE/0373). Collection of tissue was consented, and tissues were stored and used in accordance with the Human Tissue Act, United Kingdom, under local ethics approval (REC 13/NE/0373).

### Data availability.

The data that support the findings of this study are available in the Supporting Data Values or from the corresponding author upon request.

## Author contributions

MB, GCKW, AB, JFG, TB, and LAL designed research. MB, GCKW, AB, JFG, AH, KCW, LAW, and JO performed research and analyzed the data. CGB and CP provided biopsy samples. DMR oversaw mouse drug administration. TB developed the Markov model. MB wrote the manuscript, and LAL revised it. MB and GCKW contributed equally to this work; the order of co–first authors was assigned on the basis of their contributions to and preparation of the manuscript.

## Supplementary Material

Supplemental data

Unedited blot and gel images

Supporting data values

## Figures and Tables

**Table 1:**
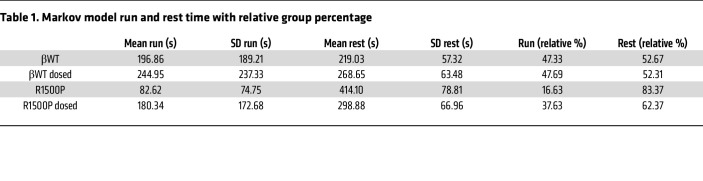
Markov model run and rest time with relative group percentage

## Version 1 05/01/2024

Electronic publication
