# Supplementary material for: A Laing distal myopathy–associated proline substitution in the β-myosin rod perturbs myosin cross-bridging activity
Source: J Clin Invest. 2024 May 1;134(9):e172599. doi: 10.1172/JCI172599 (PMC11060730; doi:10.1172/JCI172599)
Supplement: Supplemental data [file jci-134-172599-s014.pdf]

Supplemental Data

A Laing distal myopathy-associated proline substitution in the β-myosin rod perturbs myosin cross-bridging activity

Massimo Buvoli , Genevieve C. K. Wilson, Ada Buvoli, Jack Gugel, Abbi Hau, Carsten G. Bönnemann, Carmen Paradas, David M. Ryba, Kathleen C. Woulfe, Lori A. Walker, Tommaso Buvoli, Julien Ochala and Leslie A. Leinwand

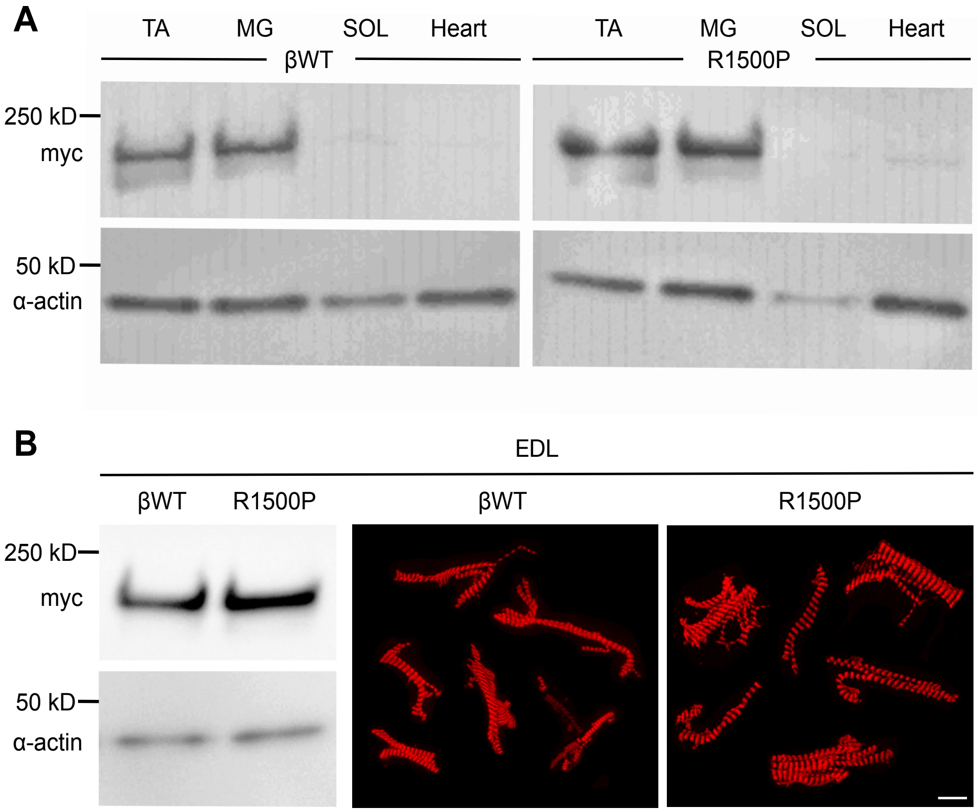

Supplemental Figure 1. Analysis of βWT and R1500P myosin expression. (**A**) Representative Western blot analysis of total protein extracts purified from 3 month-old male tibialis anterior (TA), medial gastrocnemius (MG), soleus (SOL) and heart muscles probed with myc and α-sarcomeric actin antibodies, the latter used as a loading control. (**B**) Left: western blot analysis of transgene expression in EDL muscle probed with myc and α-sarcomeric actin antibodies. Right: myofibrils from EDL muscles prepared from 6 month-old male βWT and R1500P mice immuno-stained with the myc-tag antibody. Samples were imaged on a confocal spinning disk microscope (Nikon; 100X silicon immersion objective). Bar corresponds to 5 μm.

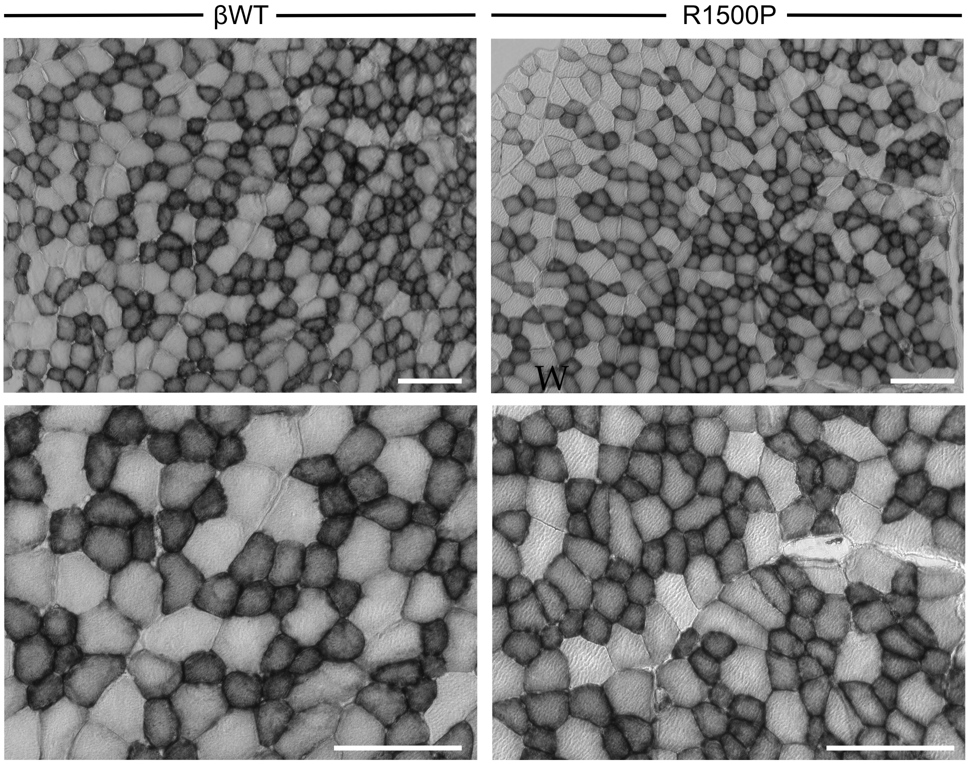

Supplemental Figure 2. Representative images of TA muscle sections used for CSA. Sections were prepared from 8-month-old male mice. Top and bottom bars correspond to 100 and 200 μm respectively. Images shown in Figure 1C were also used for CSA determination.

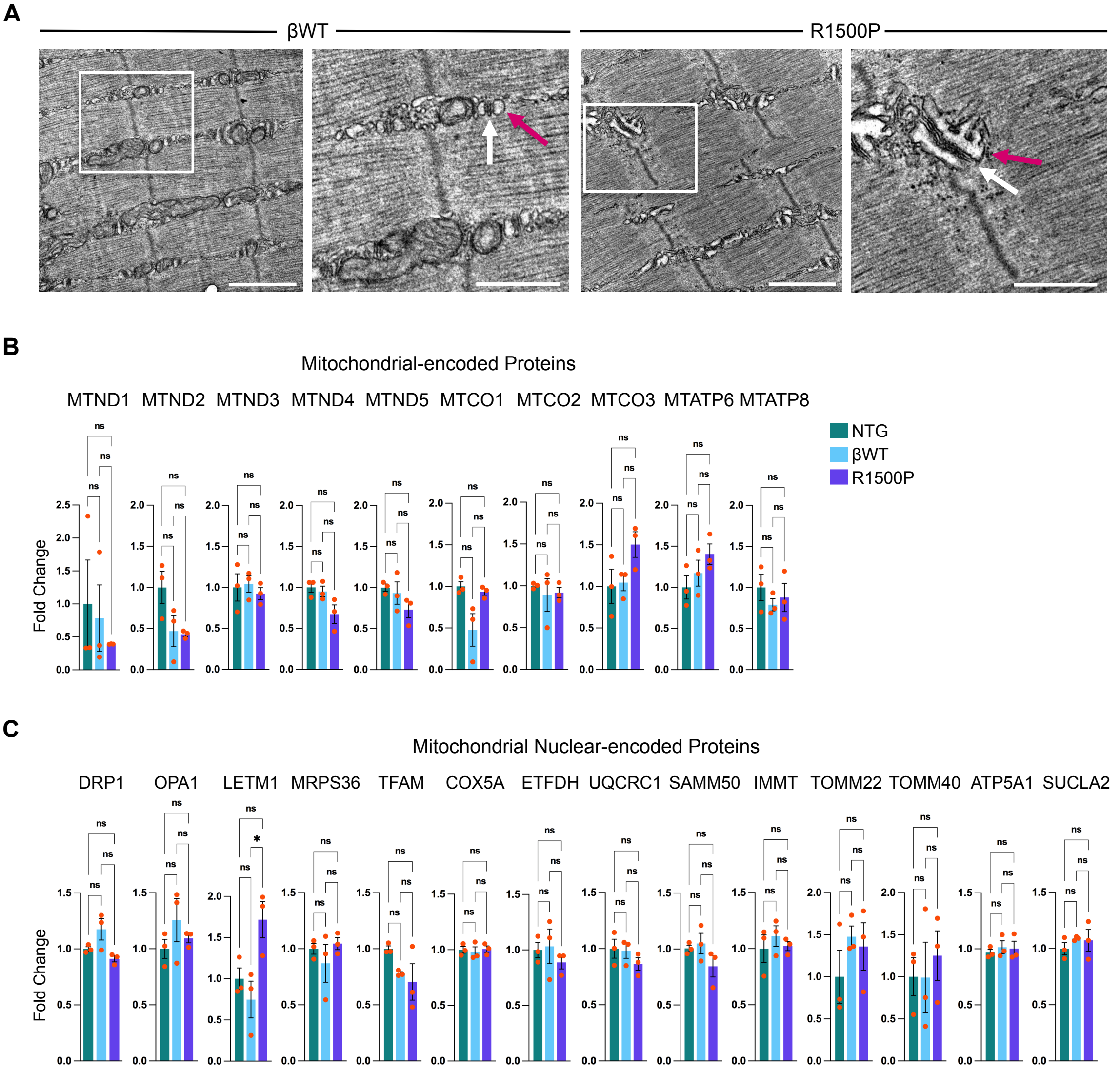

Supplemental Figure 3. Electron microscopy analysis and mitochondrial proteomics of transgenic βWT and R1500P TA muscles. (**A**) Representative electron microphotographs of longitudinal sections of TA muscles isolated from 8-month-old male βWT and R1500P mice. White boxes delimit the areas shown at higher magnification on the right side with arrows denoting a triad structure formed by T-tubules (white arrows) and two portions of the SR (magenta arrows) normally located between sarcomeres near the Z-lines. Bars correspond to 0.75 and 1 µm. (**B**) Proteomics survey of mitochondria-encoded proteins. **MTND1-5, MTCO1-3**: component of the electron-transfer chain; **MTATP6, MTATP8**: ATP synthesis. (**C**) Proteomics survey of mitochondrial nucleus-encoded proteins. **DRP1**: fission; **OPA1**: fusion; **LETM1**: morphology; **MRPS36**: translation; **TFAM**: transcription; **COX5A, ETFDH, UQCRC1**: electron-transfer chain; **SAMM50**: assembly of electron-transfer chain complexes & cristae structure maintenance; **IMMT**: cristae morphology & inner membrane architecture; **TOMM22**: Sorting and Assembly Machinery (SAM); **TOMM40**: Translocase of the Outer Membrane complex (TOM); **ATP5A1:** ATP synthesis; **SUCLA2**: succinate to succinyl-CoA conversion. 7-8 month-old male mice (n = 3/mouse group). All data are expressed as mean +/- SEM; *$p < 0.05$ by one-way ANOVA with Bonferroni multiple comparison *post hoc* test.

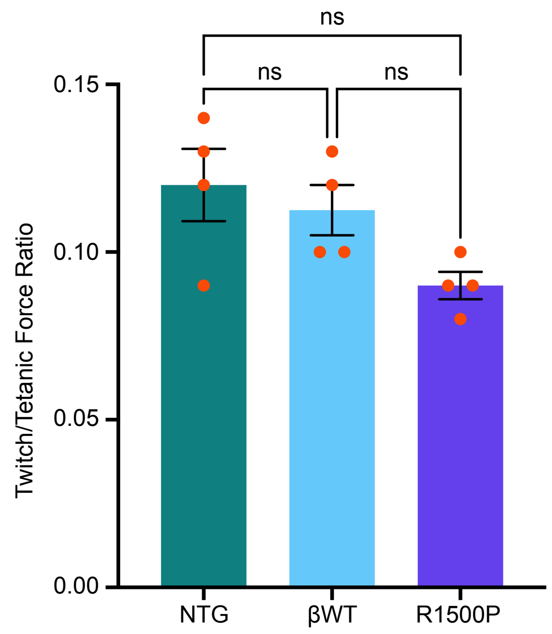

Supplemental Figure 4. ***Ex vivo*** **analysis of transgenic mouse muscles.** Ratio of βWT and RT1500P specific twitch and tetanic force 6-month-old male mice; (n = 4/mouse group).

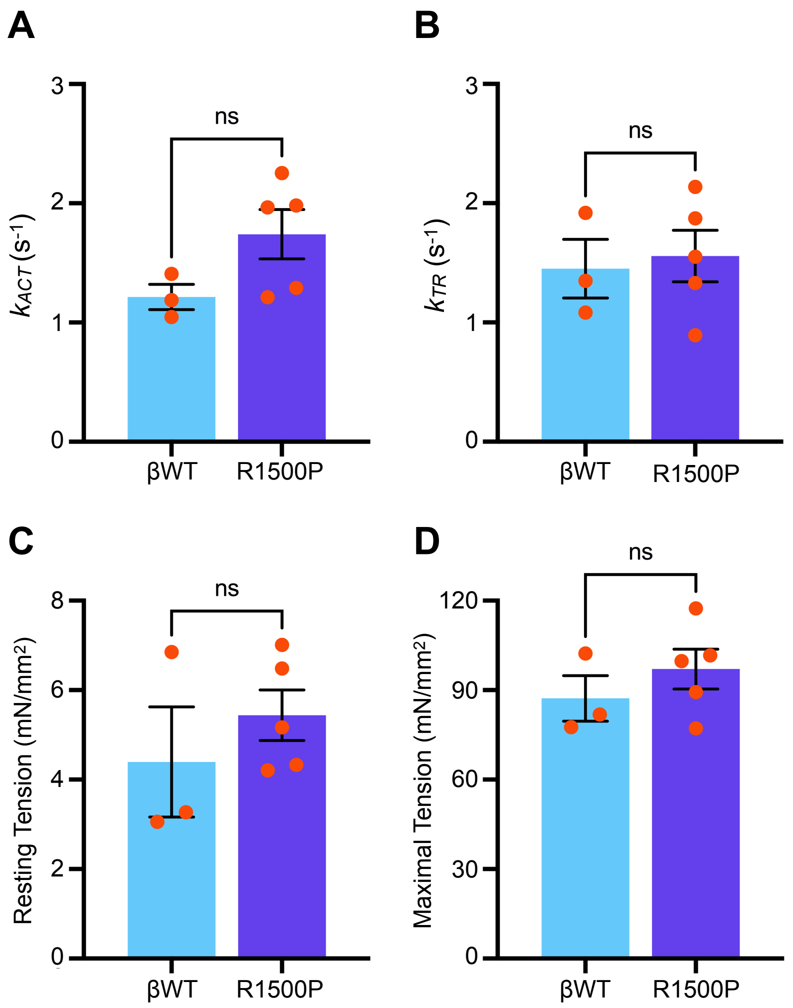

Supplemental Figure 5. βWT and R1500P myofibrils comparison of activation kinetics, resting and maximal tension. (**A**) Rate constant of tension development (k_{ACT}); (**B**) rate constant of tension redevelopment following release-restretch (k_{TR}); (**C-D**) resting and maximal tension measured from βWT and R1500P myofibrils. Myofibrils were isolated from TA muscles of 6-month-old βWT and R1500P mice. The activity of 6-8 myofibrils/muscle was averaged: βWT (*n* = 3), R1500P (*n* = 5).

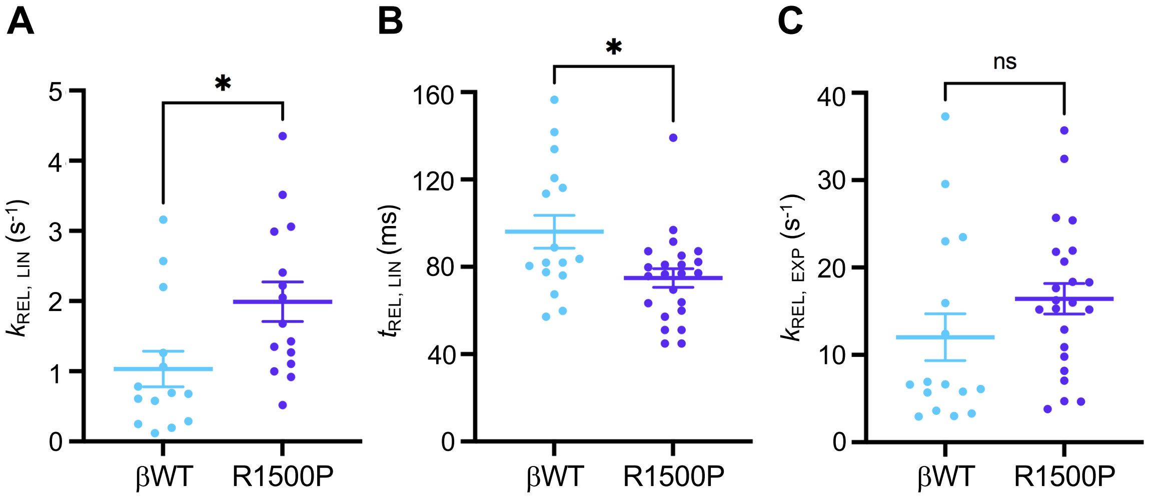

Supplemental Figure 6. βWT and R1500P individual myofibril relaxation kinetics. (**A**) Rate constant ($k_{\text{REL, LINEAR}}$); (**B**) duration of the early linear phase of relaxation ($t_{\text{REL, LINEAR}}$); (**C**) rate constant of the final exponential phase of force decline ($k_{\text{REL, EXP}}$) measured from βWT and R1500P myofibrils. 6-month-old male mice; βWT (*n* = 3), R1500P (*n* = 5). Data are expressed as mean +/- SEM; *p < 0.05, **p < 0.01 by two-tailed unpaired t-test with Welch's correction.

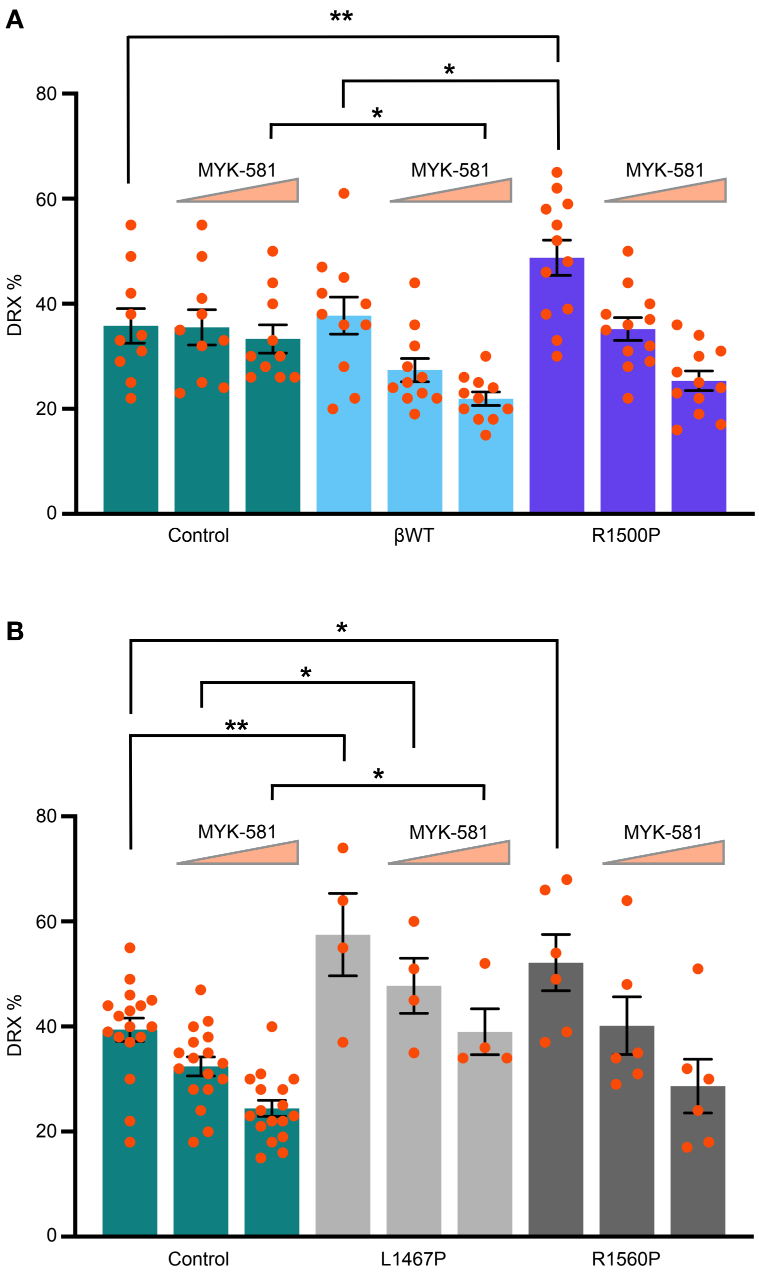

Supplemental Figure 7. Scatter plots of of myosin molecules in disorder-relaxed state (DRX) measured by Mant-ATP chase analysis. (**A**) Analysis carried out on muscle fibers purified from 6-month-old male βWT and R1500P mice. (**B**) Analysis carried out from human biopsies of patients with distal myopathy caused by the MYH7 missense mutations L1467P (35-year-old female) and R1560P (52-year-old male). Controls correspond to the mouse tibialis anterior and human vastus lateralis muscles lacking or having type 1 fibers respectively. Measurements were recorded in the absence or presence of MYK-581 (0.3 μM and 1 μM). For (**A**): Control mice (*n* = 10), βWT mice (*n* = 11), R1500P mice (*n* = 12). For (**B**): Control mice (*n* = 17), L1467P (*n* = 4), R1560P (*n* = 6). Data are expressed as mean +/- SEM; *p < 0.05, **p < 0.01 by two-way ANOVA (genotype x drug) with Bonferroni multiple comparison *post hoc* test.

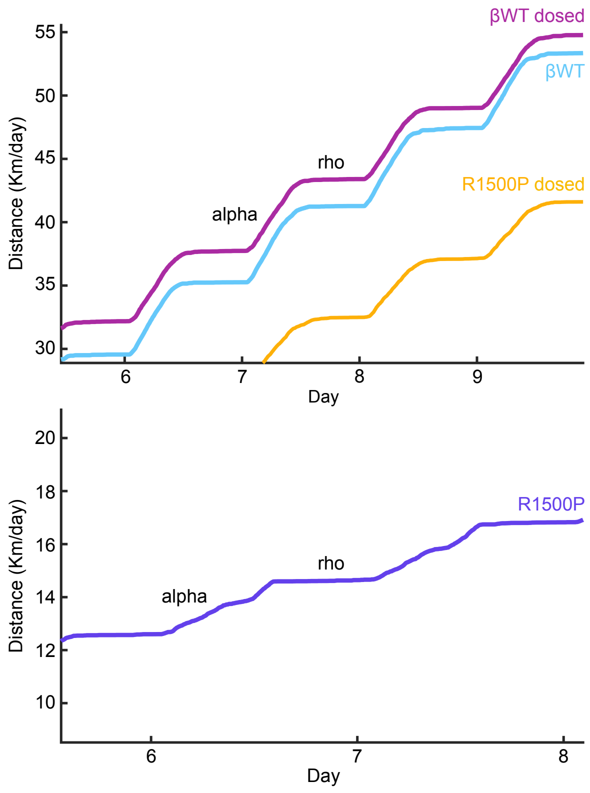

Supplemental Figure 8. Circadian phases of βWT and R1500P transgenic mice. Magnification of a section of the distance plot shown in Figure 7 (top Panel ~5X; bottom Panel ~10X). Circadian activity (alpha) and rest (rho) phases are defined.

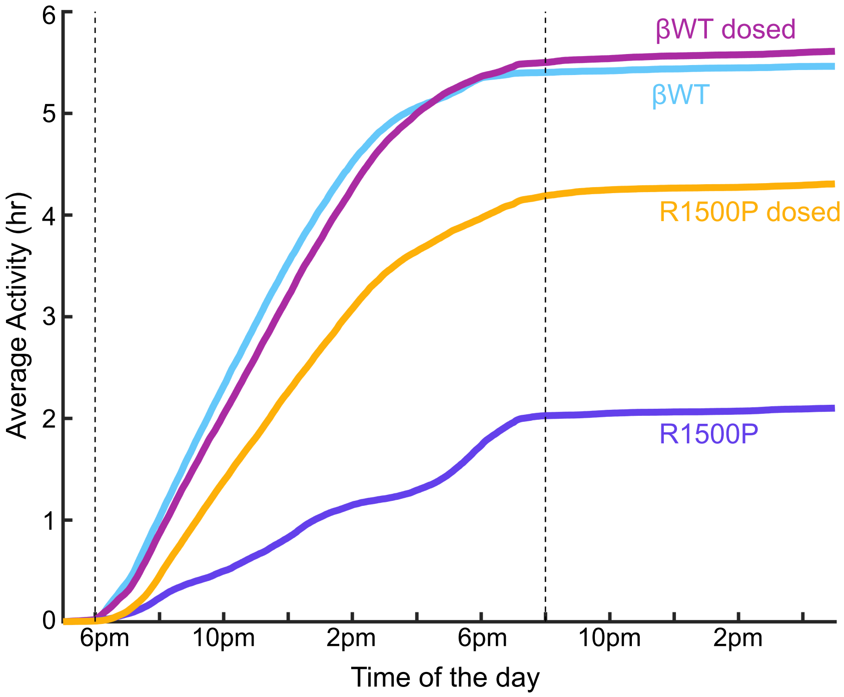

Supplemental Figure 9. **Data selection for Markov Model.** For every mouse in each group, activity sequence data were recorded during the last 6 days of the training period between 6pm-8am. Data were then combined to produce a single activity sequence used as experimental data set for the Markov model. The comparison between the activity sequence of each group is shown.

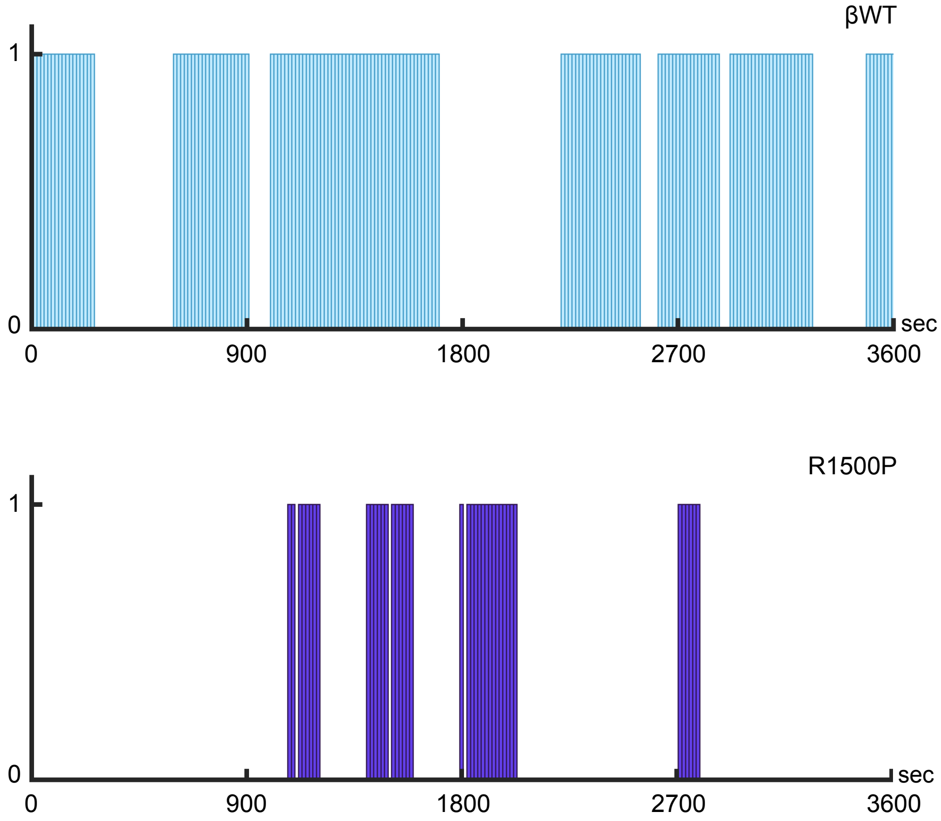

Supplemental Figure 10. Representative examples of input data used for the Markov model after binary conversion. Each mouse activity sequence is defined by the values 1 and 0 corresponding to running or resting states, respectively, measured during a 15 second period. For example, the sequence 001100 represents a mouse resting for 30 sec, then running for 30 sec and finally resting for another 30 sec. This activity sequence does not contain information about velocity and solely defines mouse running/resting time.

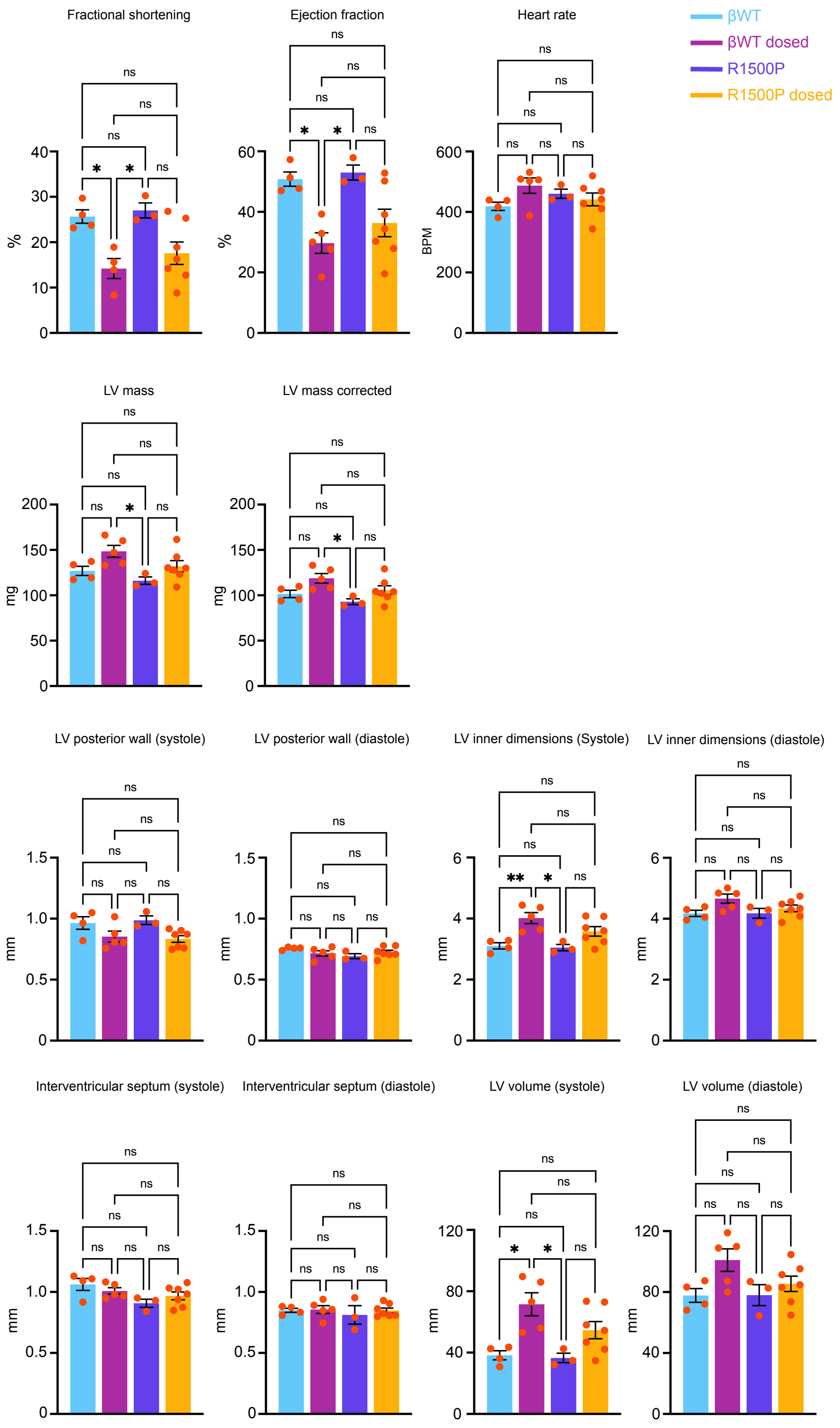

Supplemental Figure 11. Echocardiographic assessment of βWT and R1500P mouse cardiac function. At the end of the training period on the running wheel, each mouse group was subjected to echocardiographic characterization of heart contractile function. Echocardiographic parameters are reported on top of each graph. 6-month-old male mice; βWT (n = 4), βWT dosed (n = 5), R1500P (n = 3), R1500P dosed (n=7). *$p < 0.05$, **$p < 0.01$ by one-way ANOVA with Bonferroni multiple comparison *post hoc* test.

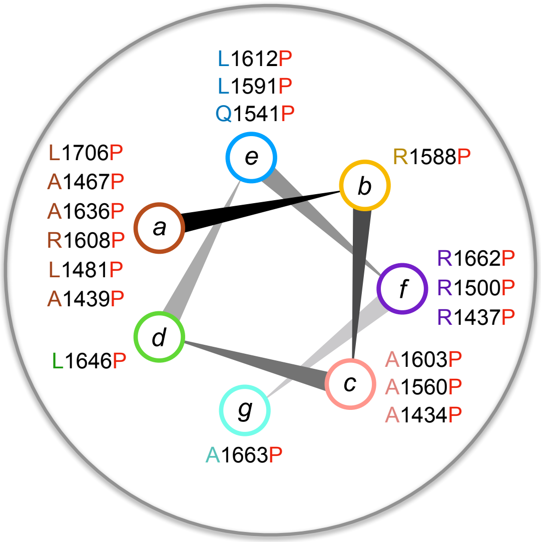

Supplemental Figure 12. Helical wheel diagram illustrating the location of MPD1 proline substitutions mapped in the MYH7 coiled-coil domain. The proline mutations are reported along the seven positions of the wheel heptad repeat color-coded and labeled *a* through *g* (7, 71). L1467P is a novel mutation not previously described (see Materials and Methods).

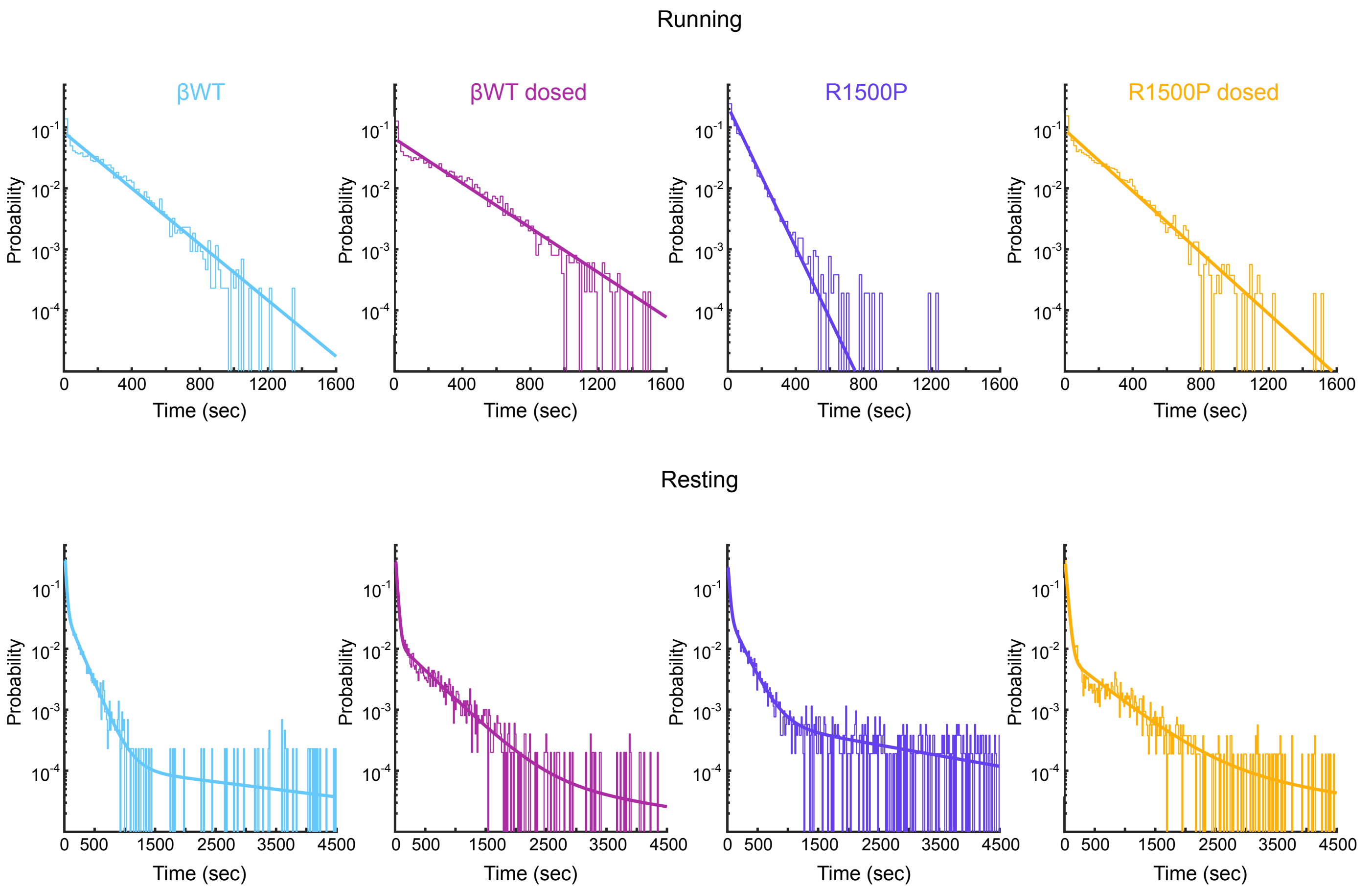

Supplemental Figure 13. Overlay of probability mass functions shown in Figure 7C and D (smooth curves) and probability mass functions generated from the raw experimental data. The close fit between the experimentally observed PMFs and Markov PMFs (See Supplemental Table 2) suggest that the statistics of mouse running bouts are well described by a power law distribution Prob(run bout lasting 15k sec) = $\alpha\beta^k$, while mouse resting bouts are well described by a linear combination of three power laws Prob(rest bout lasting 15k sec) = $\sum_{i=1}^{3} \alpha_i \beta_i^k$.

Markov run PMF:

Prob(run bout lasting 15k sec) = $\alpha\beta^{[k-1]}$ for k ≥ 1, $\alpha = 1 - prr$, and $\beta = p_{rr}$.

Markov rest PMF:

Prob(rest bout lasting 15k sec) = $\sum_{i=1}^{3} \alpha_i \, \beta_i^{[k-1]}$ for k ≥ 1 and:

$$\alpha_1 = \frac{p_{rs}(1-p_{ss})}{p_{rs}+p_{rm}+p_{rl}} \qquad \alpha_2 = \frac{p_{rm}(1-p_{mm})}{p_{rs}+p_{rm}+p_{rl}} \qquad \alpha_3 = \frac{p_{rl}(1-p_{ll})}{p_{rs}+p_{rm}+p_{rl}}$$

$$\beta_1 = p_{ss} \qquad \beta_2 = p_{mm} \qquad \beta_1 = p_{ll}.$$

Supplemental Table 1. Group distance, activity and average speed

	Distance (Km/day)			Activity (hr/day)			Avg Speed (Km/hr)		
	Cumul	Day 1	Day 15	Cumul	Day 1	Day 15	Cumul	Day 1	Day 15
βWT	8.86	4.63	10.76	5.46	4.15	5.51	2.00	1.12	2.00
βWT dosed	8.95	5.45	11.04	5.60	5.06	6.27	1.78	1.10	1.78
R1500P	1.73	1.51	1.77	2.09	1.99	2.07	0.85	0.70	0.85
R1500P dosed	4.64	4.23	6.90	4.30	3.83	4.73	1.48	1.12	1.48

Supplemental Table 2. Experimental run and rest data and relative group percentage

	Mean Run (sec)	SD Run (sec)	Mean Rest (sec)	SD Rest (sec)	Run (rel %)	Rest (rel %)
βWT	196.82	181.35	219.03	57.32	47.34	52.66
βWT dosed	244.91	224.66	268.65	63.48	47.69	52.31
R1500P	82.61	88.67	414.10	78.81	16.63	83.37
R1500P dosed	180.34	174.68	298.88	66.96	37.63	62.37

Red numbers indicate the discrepancy with the Markov model shown in Table1
