# Supplementary figures and images for: A Laing distal myopathy–associated proline substitution in the β-myosin rod perturbs myosin cross-bridging activity
Source: J Clin Invest. 2024 May 1;134(9):e172599. doi: 10.1172/JCI172599 (PMC11060730; doi:10.1172/JCI172599)

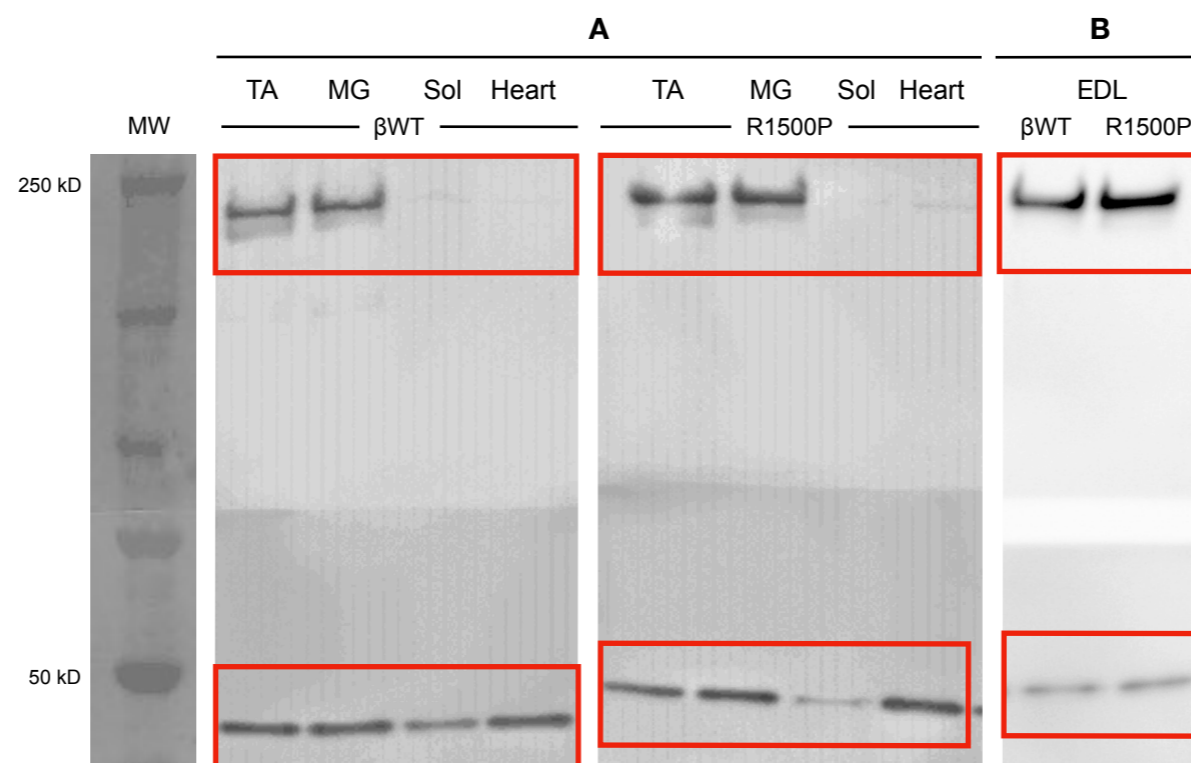

Full unedited blot/gel for Supplemental Figure 1A and B

Supplement: Unedited blot and gel images [file jci-134-172599-s015.pdf]
